# Coordinated transcriptional regulation by thyroid hormone and glucocorticoid interaction in adult mouse hippocampus-derived neuronal cells

**DOI:** 10.1371/journal.pone.0220378

**Published:** 2019-07-26

**Authors:** Pia D. Bagamasbad, Jose Ezekiel C. Espina, Joseph R. Knoedler, Arasakumar Subramani, Ariel J. Harden, Robert J. Denver

**Affiliations:** 1 Department of Molecular, Cellular and Developmental Biology, The University of Michigan, Ann Arbor, Michigan, United States of America; 2 National Institute of Molecular Biology and Biotechnology, University of the Philippines Diliman, Quezon City, Philippines; 3 Neuroscience Graduate Program, The University of Michigan, Ann Arbor, Michigan, United States of America; University of Lübeck, GERMANY

## Abstract

The hippocampus is a well-known target of thyroid hormone (TH; e.g., 3,5,3’-triiodothyronine—T_3_) and glucocorticoid (GC; e.g., corticosterone—CORT) action. Despite evidence that TH and GC play critical roles in neural development and function, few studies have identified genes and patterns of gene regulation influenced by the interaction of these hormones at a genome-wide scale. In this study we investigated gene regulation by T_3_, CORT, and T_3_ + CORT in the mouse hippocampus-derived cell line HT-22. We treated cells with T_3_, CORT, or T_3_ + CORT for 4 hr before cell harvest and RNA isolation for microarray analysis. We identified 9 genes regulated by T_3_, 432 genes by CORT, and 412 genes by T_3_ + CORT. Among the 432 CORT-regulated genes, there were 203 genes that exhibited an altered CORT response in the presence of T_3_, suggesting that T_3_ plays a significant role in modulating CORT-regulated genes. We also found 80 genes synergistically induced, and 73 genes synergistically repressed by T_3_ + CORT treatment. We performed *in silico* analysis using publicly available mouse neuronal chromatin immunoprecipitation-sequencing datasets and identified a considerable number of synergistically regulated genes with TH receptor and GC receptor peaks mapping within 1 kb of chromatin marks indicative of hormone-responsive enhancer regions. Functional annotation clustering of synergistically regulated genes reveal the relevance of proteasomal-dependent degradation, neuroprotective effect of growth hormones, and neuroinflammatory responses as key pathways to how TH and GC may coordinately influence learning and memory. Taken together, our transcriptome data represents a promising exploratory dataset for further study of common molecular mechanisms behind synergistic TH and GC gene regulation, and identify specific genes and their role in processes mediated by cross-talk between the thyroid and stress axes in a mammalian hippocampal model system.

## Introduction

Thyroid hormone (TH_;_ e.g., 3,5,3’- triiodothyronine—T_3_) and glucocorticoids (GCs; stress hormones; e.g., cortisol and corticosterone) are known to influence neuronal processes such as learning and memory, in part through their actions on the hippocampus. Deficiencies in TH during neonatal or postnatal life result in morphological and functional changes in the hippocampus, leading to delayed neurogenesis and neuronal maturation in key regions such as the dentate gyrus, and consequent impairment in behavioral and cognitive functions associated with this brain region [1–7]. Glucocorticoids have also been shown to influence hippocampal development and other hippocampal-related cognitive functions but in a context-dependent manner. Chronic stress or prolonged exposure to high GC levels results in an impairment in learning and memory formation in rodents through the promotion of neuronal atrophy and disruption of inter-neuronal connections in the hippocampus [8–12]. Conversely, acute moderate stress has been shown to enhance memory consolidation [13–15], and alter long-term potentiation and synaptic plasticity depending on subfield of the hippocampus [16]. Selectively blocking GC action in mouse hippocampal neural progenitor cells results in altered neuron developmental patterns that is accompanied by impaired memory consolidation, emphasizing the importance of GC action in the hippocampus [17].

Thyroid hormones and GCs exert their effects by binding to their cognate nuclear hormone receptors (NRs), a class of ligand-activated transcription factors [18, 19]. Glucocorticoids bind to the GC receptor (GR), which is localized to the cytoplasm in the absence of ligand [20, 21]. Upon ligand binding, GR forms a homodimer that then translocates to the nucleus and binds to GC response elements (GRE) in DNA. Once bound to GREs, GRs recruit other transcription factors and co-regulators that alter the chromatin environment to either up-regulate or down-regulate target gene expression [20, 21]. In contrast to GRs, the TH receptor (TR) in the absence of ligand is bound to DNA at TH response elements (TRE) as a heterodimer with retinoid X receptor (RXR) complexed with co-repressors and histone deacetylases, resulting in a closed chromatin structure [22]. Upon ligand binding, TR changes conformation, corepressor complexes are released, and coactivator complexes are recruited resulting in an open chromatin structure that is more accessible to transcription factors and RNA polymerase 2 (Pol2) [22]. In addition to this classical transcriptional mechanism mediated by the TR, more recent evidence has demonstrated that ligand binding can modulate the binding affinity of TR to its target motifs [23], and that TR binding is dynamic, and may increase in the presence of ligand [24]. Negative gene regulation by TH may occur through a decrease in TR recruitment in the presence of ligand, or through mechanisms that do not involve direct TR binding [23]. The GREs and TREs of hormone responsive genes can be found both at promoters and at regions distal to the promoters that interact with the transcription complex via long-range chromatin looping and protein-protein interactions through the action of the Mediator (Med) complex and other transcriptional co-regulators [25–27].

Although it is well established that TH and GCs influence the structure and function of hippocampal neurons [28–30], few studies have investigated the interaction between, and combined effects of TH and GC [31]. Several gene expression analyses have been conducted to identify GC-regulated genes in hippocampal neurons [32–36]. Some have examined the cross-talk between TH and GC signaling in regulating the expression of a select number of genes in mouse primary cerebrocortical cells [37]. Another study explored the interaction between the adrenal and thyroid axis in the context of offspring-parent interactions where maternal care was found to increase T_3_ levels, which consequently increased GR transcription in the hippocampus of the offspring [38]. A study by Kulkarni and Buchholz (39) conducted genome-wide expression analysis to investigate the gene regulatory cascades regulated by TH and GC that drive tadpole tail resorption and metamorphosis. However, despite the well-established influence of TH and GC in shaping neuronal morphology and function, to our knowledge no large-scale gene expression analysis studies have been done on any mammalian cell type to identify genes that are coordinately regulated by TH and GC. Earlier, we found that the Krüppel-like factor 9 (*Klf9*) gene, which encodes a transcription factor known to influence neuronal structure and function [40–44], to be independently [45–48] and synergistically regulated by TH and GC in mouse hippocampus and in the adult mouse hippocampus-derived cell line HT-22 [49]. However, thus far *Klf9* is the only gene known to be regulated in this manner in the hippocampus. We therefore sought to determine if the synergistic regulation of *Klf9* was an isolated phenomenon, or if there are other genes that are similarly regulated. If other genes show similar synergistic induction by hormones, this might point to similar transcriptional mechanisms, and perhaps related functions in hormone action on hippocampal cells.

## Materials and methods

### Cell culture

The HT-22 cell line was obtained from Dr. David Schubert of The Salk Institute, La Jolla. HT-22 is a cell line derived from mouse hippocampus immortalized with the SV40 T antigen [50, 51]. This cell line exhibits properties of differentiated neurons; e.g., they express neuron specific markers such as enolase and the neurofilament triplet, but not the glial cell marker, glial fibrillary acidic protein [50–53]. HT-22 cells have been previously shown to express functional TR and GR [46, 49]. Cells were cultured in Dulbecco’s modified Eagle’s medium (DMEM; Invitrogen) supplemented with sodium bicarbonate (2.2 g/L), penicillin G (100 units/mL), streptomycin sulfate (100 μg/mL) and 10% fetal bovine serum (FBS) that had been stripped of thyroid [54] and steroid hormones [55]. Cells were cultured under a humidified atmosphere of 5% CO_2_ at 37°C. For microarray-based gene expression analysis we seeded cells at a density of 1 x10^7^ cells per well in 100 cm^2^ plates.

For validation of gene expression by RTqPCR, cells were seeded at a density of 2.5 x 10^6^ cells per well in 6-well plates. When cells reached ~90–95% confluency, and immediately before hormone treatments, we replaced the growth medium with serum-free DMEM supplemented with 30 nM of 3,5,3’-triiodothyronine (T_3_; Sigma T2752) dissolved in dimethylsulfoxide (DMSO), 100 nM Corticosterone (CORT; Sigma C2505) dissolved in 100% ethanol, or 30 nM T_3_ + 100 nM CORT. All experiments received the same volume of DMSO (0.03%) and ethanol (0.001%). To identify synergistic interactions, hormone concentrations were based on submaximal dose previously determined from dose response assays [49]. Control treatments received an equivalent concentration of vehicle (0.03% DMSO and 0.001% ethanol). All hormone treatments were continued for 4 hr before cell harvest. Each hormone treatment was performed in triplicate for RNA extraction and microarray analysis.

### RNA extraction, reverse transcription and quantitative PCR

We extracted total RNA from HT-22 cells using the Trizol reagent (Invitrogen) following the manufacturer’s protocol. The extracted RNA was further purified using the RNAeasy Kit (Qiagen) to obtain a UV absorbance ratio at A_260/280_ to be between 1.8–2.0. We measured *Klf9* gene expression by reverse transcription (RT)-quantitative PCR (qPCR) to verify that the hormone treatments worked before microarray analysis. For qPCR validation of the results of the microarray analysis, we treated RNA with DNase I (Sigma DNAseI Amplification Grade cat no: D5307) prior to cDNA synthesis. First strand cDNA synthesis was done using the ABI High-Capacity cDNA Reverse Transcription kit with RNase inhibitor from Applied Biosystems (Life Technologies Corp., Carlsbad, CA). Gene expression analysis was performed using qPCR SYBR Green LO ROX mix (PCR Biosystems) run in a Real-time PCR (ABI 7500 Fast). We designed oligonucleotide primers to span an exon-exon boundary for each gene analyzed (S1 Table), and we used a relative quantification method using a pool of cDNA [49]. For measuring enhancer RNA (eRNA) levels, we designed oligonucleotide primers to amplify 80–100 bp fragments from the enhancer sequences, and we included minus reverse transcriptase cDNA controls to account for possible genomic DNA contamination. Melt curve analysis was also performed to determine specificity of amplification by primers. We normalized mRNA levels to the mRNA of the reference gene *Ppia* which was unaffected by hormone treatments.

### Microarray analysis

Total RNA (175 ng per sample) was amplified using the Illumina Totalprep RNA Amplification Kit (Ambion, Inc.) to generate biotinylated amplified RNA. The biotinylated RNA (2 μg) was hybridized at 55° C for 22 hr to Illumina Bead Chips (Illumina Mouse WG-6 v2.0). Microarrays were washed and scanned for data collection as directed by the manufacturer. Microarray data was normalized (Rank Invariant), and analyzed with Illumina BeadStudio software. The mean array value of three replicates per treatment was used to compute fold change over control.

### Sorting gene lists

For filtering of the microarray data set, we calculated gene expression fold change (FC) as the ratio between the average of the hormone treatment hybridization signal intensity (n = 3/hormone treatment) and average of the vehicle control treatment hybridization intensity. The Log_2_ of the gene expression FC was also calculated. Hormone-dependent regulation was identified based on a cut-off *P* value of 0.02 and a 1.5 FC (Log_2_FC = 0.58496) compared to control for T_3_, CORT, and T_3_ + CORT-treated samples. Additional gene lists comparing differences between T_3_ + CORT and T_3_ alone or CORT alone were also generated at a *P* value cutoff of 0.02. These criteria represent a more conservative estimate of genes which are hormone regulated and we did this to minimize false positives being called out. We have deposited our microarray dataset in GEO (GSE132423) to make the data accessible to those who may want to use a less stringent approach for gene discovery. To cluster the different genes, we generated Venn diagrams comparing the gene lists for T_3_-regulated, CORT-regulated, and T_3_ + CORT regulated genes. Two-color heatmap was also generated using the average array signal values per treatment replicate of the differentially expressed genes on the web-based tool Morpheus (Morpheus, https://software.broadinstitute.org/morpheus/). The most intense blue color represents the lowest row value while the most intense red color represents the highest row value. We performed additional clustering analysis on the intersections of the three gene lists to further segregate the genes into different patterns of gene regulation similar to the method described by Kulkarni and Buchholz (39). For this, we determined if the T_3_ + CORT microarray values of the genes found in the intersections were significantly different from T_3_ or CORT alone.

Genes are considered to be synergistically regulated by T_3_ + CORT if: 1) there is no effect with T_3_ and CORT alone but a statistically significant effect with T_3_ + CORT treatment based on *P*<0.02 and 1.5 FC, or 2) an effect with combined T_3_ + CORT treatment that is greater than the additive effect of T_3_ and CORT alone by one standard deviation [56]. Whether the combined effect of T_3_ + CORT on gene expression was greater than the additive effect of either hormone alone was determined by unpaired Welch’s t-test, which compared the combined hormone treatment array signal to the sum of the T_3_ alone and the CORT alone array signals. Variance of the combined array signal was calculated as the sum of the variances of the signals from each hormone treatment alone.

### *In silico* mapping of potential transcription factor binding sites and open chromatin marks

We accessed publicly available chromatin immunoprecipitation (ChIP)-seq data for GR conducted on rat hippocampus [57, 58], TR conducted on the immortalized mouse cerebellar neuronal C17.2 cell line [59], Med1/12 ChIP-seq and RNA Pol2 ChIP-seq conducted on mouse embryonic stem cells and fibroblasts [27], and H3K27Ac ChIP-seq conducted on terminally differentiated neural progenitor cells [60]. We used the BedOPs tool [61] to map these data to the regions flanking genes that we found to be synergistically regulated by T_3_ + CORT in HT-22 cells, and to a random set of 300 genes that were not differentially regulated in any of the hormone treatment. We set our search parameters to include the gene coding region and 50 kb flanking the transcription start and termination site. These parameters were set based on previous findings that (i) TR binding sites were found to be highly enriched within coding regions [23], (ii) CORT induced DNAse I hypersensitive sites were found to be enriched within ± 50kb of GR-responsive genes [62], and (iii) transcription factor binding, including TR and GR, is enriched at enhancers which may be located far from gene promoters [63–65]. The Mm10 genome assembly coordinates of the synergistically regulated genes were obtained using UCSC’s table browser tool [66]. Coordinates from rat ChIP-seq data, and older mouse assembly coordinates were converted to the Mm10 assembly using UCSC’s liftOver utility [67]. Candidate transcription factor binding sites for GR and TR were predicted using the Length-Aware Site Alignment Guided by Nucleotide Association (LASAGNA) algorithm [68]. Potential GREs were predicted using the vertebrate GR model and potential TREs were predicted using the *Rattus norvegicus* T_3_R-β and T_3_R-α models.

### Functional annotation clustering

For functional annotation clustering, we generated gene lists comprising genes synergistically regulated by combined hormone treatment (T_3_ + CORT), genes regulated by T_3_ alone, and genes regulated by CORT alone. Genes which had duplicate probe sets within each gene list were collapsed into single entries. Each gene list was submitted as a list of Entrez gene IDs to the Database for Annotation, Visualization and Integrated Discovery (DAVID) v6.8 for functional annotation analysis [69, 70]. Genes that did not have mappable Entrez IDs were excluded from the analysis. Functional annotation clustering was run with the following parameters: Kappa similarity–Similarity term overlap: 3, Similarity threshold: 0.50; Classification–Initial group membership: 3, Final group membership: 3, Multiple linkage threshold: 0.50; Enrichment thresholds–EASE: 1.0. These parameters correspond to a “Medium” level of stringency to eliminate most false positives but to still obtain useful functional annotation clusters. A higher stringency analysis was performed but did not yield any functional annotation clusters, likely due to the limited dataset that we had.

### Plasmid constructs and transient transfection assay

Using genomic DNA extracted from HT-22 cells, we PCR-amplified an 850 bp DNA fragment corresponding to a predicted upstream enhancer located 9.9 kb upstream of the transcription start site (TSS) of the mouse *Cytochrome b561* (m*Cyb561*) gene, and a 400 bp DNA fragment corresponding to a predicted intronic enhancer within intron 3 of the m*Cyb561* gene. The m*Cyb561* upstream enhancer was subcloned into the pGL4.23 promoter-*Firefly* luciferase reporter (Promega) vector at the SacI and BglII sites, and the m*Cyb561* intronic enhancer was subcloned at the SacI and XhoI sites to generate plasmids for transient transfection enhancer-reporter assays. We designed mutagenesis primers using the web design tool of the QuickChange Lightning Multi-site Directed Mutagenesis Kit (Agilent) or the Q5 Site-Directed Mutagenesis Kit (New England Biolabs, Inc.) to mutagenize predicted hormone response elements (HREs), and followed to the manufacturer’s protocol for PCR-based mutagenesis reactions. Primers were designed to convert the most conserved bases of the half-sites in the GREs or TREs into Ts (S2 Table).

For dual-luciferase enhancer-reporter assays, HT-22 cells were seeded at a density of 1.0 x 10^4^ cells per well in 96-well tissue culture plates in T_3_ and steroid-stripped medium. Cells were transfected at 60–70% confluency with 95 ng of the pGL4.23-m*Cyb561* enhancer constructs and 5 ng of the normalization reporter p*Renilla* luciferase- thymidine kinase construct (Promega) using the XtremeGene HP DNA transfection reagent (Roche) following the manufacturer’s protocol. Eighteen hours after transfection, hormone treatment was performed in serum-free medium with vehicle, T_3_ (30 nM), CORT (100 nM), or T_3_ + CORT for 4 hr before harvesting for dual-luciferase assay using Dual Luciferase Assay System (Promega) and the Fluoroskan FL microplate luminometer (Thermo Scientific). Enhancer-reporter assays were done twice with 4–6 replicates per treatment.

### Chromatin immunoprecipitation

We isolated chromatin from HT-22 cells treated with vehicle, T_3_ (30nM), CORT (100nM), or T_3_ + CORT. Five micrograms of sheared chromatin were used for each reaction, and ChIP assays were conducted as described by Denver and Williamson [48]. We used a rabbit polyclonal antiserum raised against the full-length *X*. *laevis* TRβ (PB antiserum, 5 μL; it does not distinguish TRα from TRβ; provided by Yun-Bo Shi). This antiserum has been used extensively for ChIP assay on frog tissues [48, 71, 72] and in mouse [73, 74]. The frog and mouse TR proteins share greater than 90% sequence identity. We also used commercial antibody to mouse GR (5 μg; M-20X; Santa Cruz Biotechnology, Inc) or normal rabbit serum (NRS) (Sigma). We analyzed ChIP DNA by quantitative real-time PCR using SYBR Green (Applied Biosystems) and primer pairs that targeted the putative *Cyb561* enhancers (S3 Table).

### Data analysis and statistics

The microarray data set was filtered with python scripts using the SciPy ecosystem [75] to generate lists of genes regulated by T_3_, CORT, and T_3_ + CORT. Venn diagram analysis of the gene lists was done with python scripts using the dataframe analysis package Pandas in the SciPy ecosystem [76]. Statistical analysis for synergistically regulated genes based on array signal intensity was done using Welch’s t-test on Microsoft Excel. Data for dual luciferase assays (*Firefly* luciferase divided by *Renilla* luciefase counts) and ChIP assays (expressed as the ratio of ChIP signal to input) were log_10_ transformed before statistical analysis. Gene expression data are reported as the mean + SEM. Luciferase, RTqPCR, and ChIP data were analyzed using one-way ANOVA followed by Tukey’s posthoc test using GraphPad Prism version 7.00 for Windows (GraphPad Software, La Jolla California USA, www.graphpad.com), and *P* < 0.05 was accepted as statistically significant.

## Results

To investigate actions of T_3_ and CORT in hippocampal neurons, we conducted gene expression analysis on HT-22 cells, an adult mouse hippocampus-derived neuronal cell line, treated with vehicle, T_3_, CORT, or T_3_ + CORT for 4 hr. To analyze the microarray data, three lists of differentially expressed genes were generated corresponding to T_3_ vs. vehicle, CORT vs. vehicle, and T_3_ + CORT vs. vehicle. Only 9 genes were differentially expressed in response to T_3_ (S4 Table), 432 genes in response to CORT (S5 Table, Table B in S1 File), and 412 genes in response to T_3_ + CORT treatment (S6 Table, Table C in S1 File).

**Fig 1:**
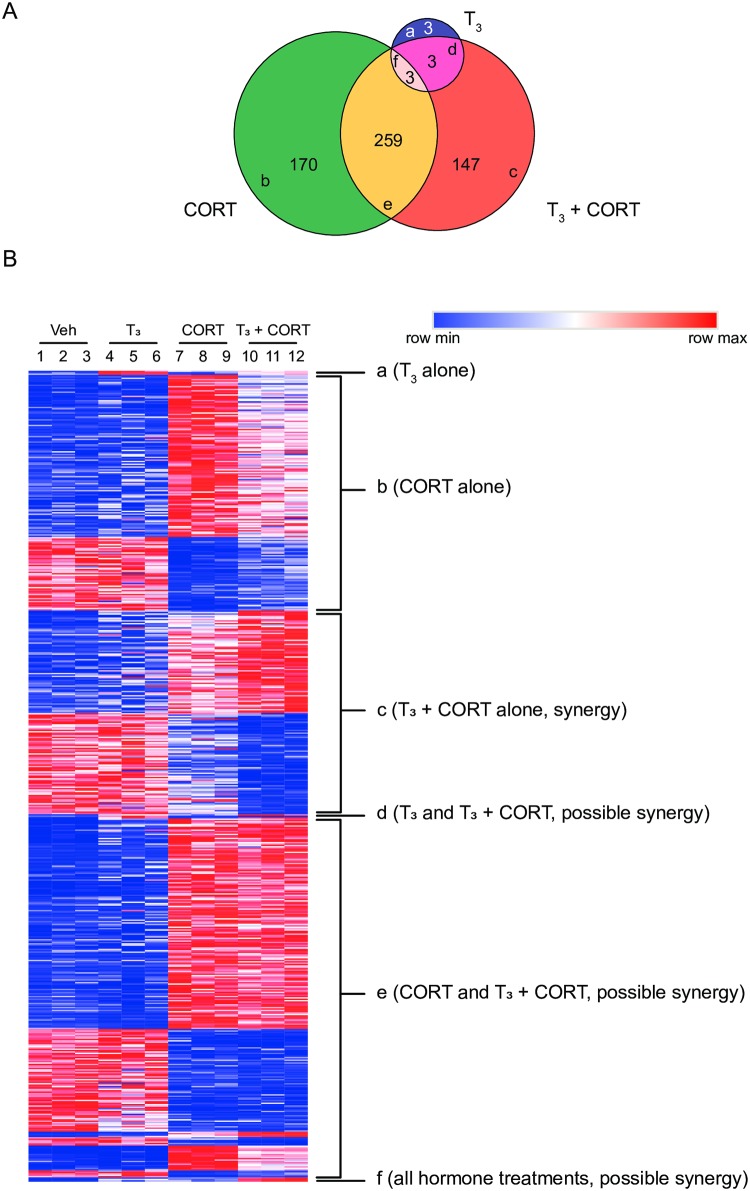
Number of genes and expression patterns of genes regulated by T_3_, CORT, and T_3_ + CORT. **(A)** A three-way Venn diagram comparing overlaps of genes differentially expressed in T_3_, CORT, and T_3_ + CORT based on a 1.5 fold-change (FC) and *P*<0.02 segregates the genes into 6 regions corresponding to different patterns of gene regulation (a-f). **(B)** Heatmap showing the relative expression of genes called as differentially regulated in T_3_, CORT, and T_3_ + CORT corresponding to sections a-f of the Venn diagram.

Using the three gene lists, a three-way Venn diagram was constructed (Fig 1A) to further parse different patterns of gene expression (Table 1) in response to hormone treatments. Additional clustering was performed to determine if the array signal of T_3_ or CORT alone regulated genes were significantly different from array signal of combined treatment with T_3_ + CORT (genes found at the intersections of the Venn diagram; Fig 1A, sections d-f). That is, genes that were additively or synergistically regulated by T_3_ + CORT.

**Table 1:** Summary of expression patterns of different gene sets.

Code	Description
A1	Differentially expressed in T_3_, CORT abolishes T_3_ response
B1, B2	Differentially expressed in CORT, T_3_ abolishes CORT response
C1, C2	Differentially expressed in T_3_ + CORT combined treatment only
D1, D2, D3	Differentially expressed in T_3_ and T_3_ + CORT
E1, E2	Differentially expressed in CORT, T_3_ treatment does not affect CORT response
E3, E4	Differentially expressed in CORT, T_3_ treatment enhances CORT response
E5, E6	Differentially expressed in CORT, T_3_ treatment suppresses CORT response
F1	Differentially expressed in all treatments

### Genes regulated by T_3_ alone

Treatment with T_3_ changed the mRNA level of 9 genes, and of these, 3 were also found in the CORT, and 3 in the T_3_ + CORT groups (Fig 1A). The three genes in section a (Fig 1A, section a, Table D in S1 File) are responsive to T_3_, but CORT treatment appears to abolish the T_3_ response.

### Genes regulated by CORT alone

Treatment with CORT changed the mRNA levels of 432 genes, and of these, 170 genes were regulated only by CORT (Fig 1A, section b). That is, of the 432 genes regulated by CORT alone, the CORT response of 170 of these genes is abolished in the presence of T_3_ (Table E in S1 File), since these genes were not found in the T_3_ + CORT treatment. Heat maps of these 170 genes are shown in Fig 1B (section b), where 117 genes were induced and 53 genes repressed.

### Genes regulated by T_3_ + CORT

There were 412 genes differentially expressed in response to combined T_3_ + CORT treatment. Of these, 147 were responsive only to combined hormone treatment (Fig 1A, section c, Table F in S1 File), and therefore fulfill the first definition of synergistic regulation. Among the 147 synergistically regulated genes, 74 were induced and 73 were repressed. As can be seen in the heatmap (Fig 1B, section c), the magnitude of increase (red) or decrease (blue) in gene expression of the synergistically regulated genes across treatments is more pronounced with combined hormone treatment than with T_3_ or CORT alone.

Genes which were called as differentially expressed in more than one hormone-treatment fall in the intersections of the Venn diagram. Genes appearing in these intersections may be considered synergistically regulated by T_3_ and CORT if the T_3_ + CORT signal is significantly greater than the computed additive effect of T_3_ and CORT array signal.

**Fig 2:**
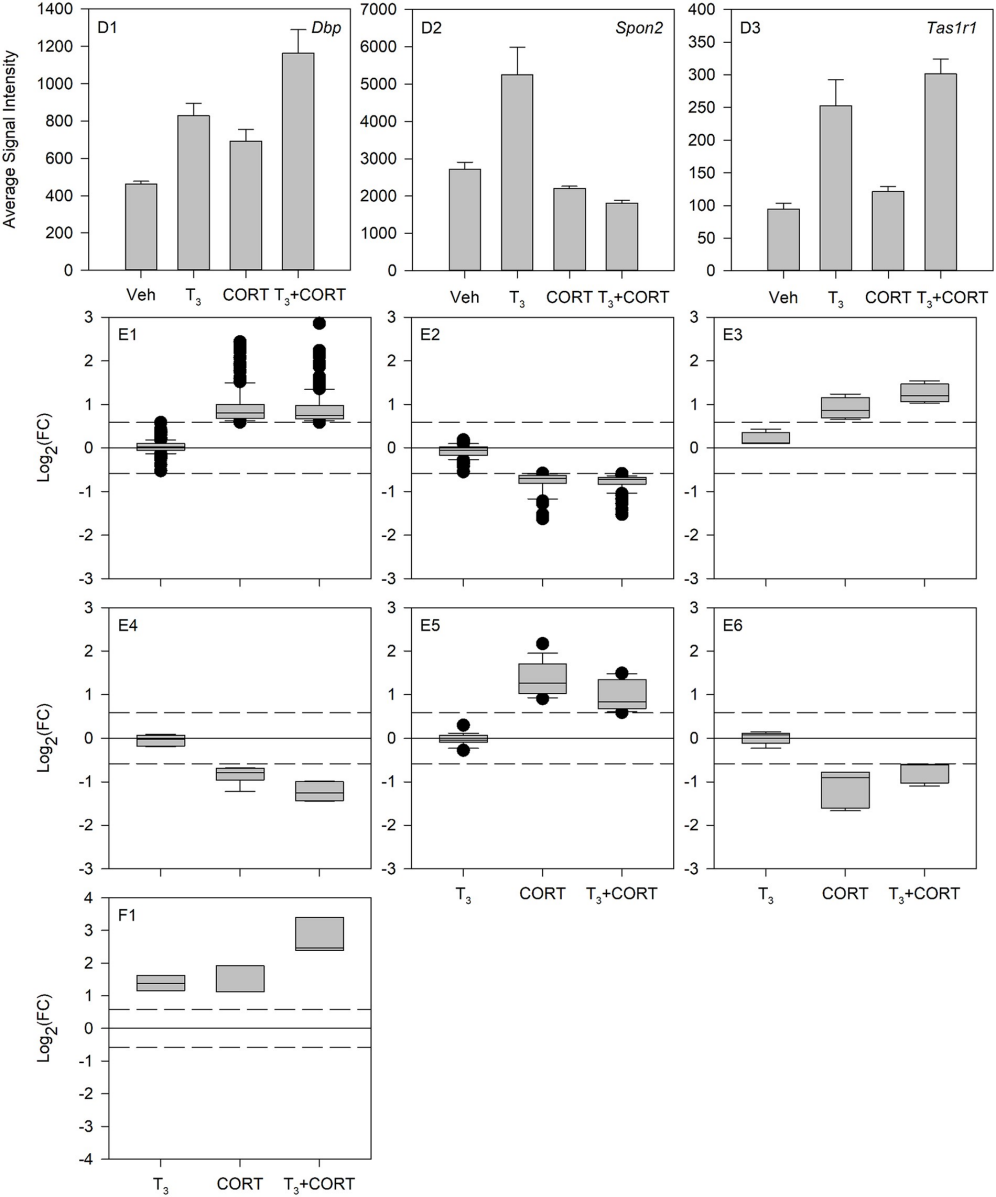
Generalized expression patterns of genes differentially expressed in more than one hormone treatment. Bar plots and box plots were created to represent the different regulation patterns of genes differentially expressed in T_3_ and T_3_ + CORT (D1-3), CORT and T_3_ + CORT (E1-6), and T_3_, CORT, and T_3_ + CORT (F1). For D1-3, gene expression is plotted in mean array signal value ± standard deviation per hormone treatment (Vehicle, T_3_, CORT, and T_3_+CORT). For E1-6 and F1, the Log_2_ of the fold-change value per gene was plotted in box plots. The box plots show the median, 10^th^, 25^th^, 75^th^, and 90^th^ percentile as vertical boxes with error bars. Black circles represent outliers in the data set. The dashed lines represent the Log_2_ of 1.5 fold-change cut-off for differential expression.

Results showed three genes (*Tas1r1*, *Dbp*, *Spon2*) that were differentially expressed in both the T_3_ and the T_3_ + CORT treatments, but not with CORT treatment alone (Fig 1A, section d). Two of these genes (*Dbp* and *Spon2*) showed statistically significant differences between the T_3_ alone and the T_3_ + CORT treatments (Table G in S1 File). *Dbp* expression was induced in response to T_3_, and this effect was enhanced by combined hormone treatment but was not synergistic (Fig 2, D1). Interestingly, *Spon2* expression was increased by T_3_, but was decreased by combined hormone treatment (Fig 2, D2). In contrast, *Tas1r1* was shown to be induced by T_3_ and this effect persisted even in the presence of CORT (Fig 2, D3, Table H in S1 File).

There were 259 genes that were differentially expressed in both the CORT and the T_3_ + CORT treatments, but not with T_3_ alone (Fig 1A, section e). Of these, 226 genes showed CORT response levels similar to that observed in the T_3_ + CORT treatment, indicating that the expression of these genes was only influenced by CORT (Fig 2, E1-2; Table 1: E1-2; Table I in S1 File). Thirty three of the 259 genes exhibited expression levels that were significantly different between the CORT alone and the T_3_ + CORT treatments. Of these 33 genes, T_3_ enhanced the effect of CORT (increase or decrease) on the expression of 10 genes (Fig 2, E3-4, Table 1: E3-4, Table J in S1 File), and 3 of these 10 showed synergy (Table K in S1 File). For the remaining 23 genes, T_3_ suppressed the CORT response in combined hormone treatment when compared to CORT treatment alone (Fig 2, E5-6; Table 1 E5-6; Table L in S1 File).

Three genes were found to have increased expression in all three hormone treatment groups (*Cyb561*, *Klf9*, *2310051E17Rik*; Fig 1A, section f; Table M in S1 File). Furthermore, these three genes were the top three most synergistically induced genes. In total, there were 80 synergistically induced (Table 2; S1 Fig) and 73 synergistically repressed (Table 3; S1 Fig) genes identified in the dataset (Table K in S1 File).

**Table 2:** Top 20 genes synergistically induced by T_3_ + CORT.

SYMBOL	T_3_ Fold Change	CORT Fold Change	T_3_ + CORT Fold Change
*Cyb561*	2.61	3.78	10.52
*Klf9*	3.09	2.17	5.52
*Ear3*	1.06	2.50	3.79
*Lims2*	1.08	2.36	2.91
*E230024B12Rik*	1.48	1.20	2.28
*Errfi1*	1.21	1.46	2.10
*B430216N15Rik*	1.41	1.18	2.02
*Lbr*	1.13	1.46	1.85
*Ubtd2*	1.15	1.43	1.74
*Sh3rf1*	1.02	1.48	1.73
*9626962_3*	1.34	1.46	1.73
*Slc3a2*	1.20	1.48	1.73
*Mcm4*	1.09	1.49	1.65
*Slc38a4*	1.11	1.46	1.65
*1110034A24Rik*	1.03	1.29	1.64
*Gprc5a*	1.37	1.28	1.63
*Psmd2*	0.97	1.42	1.63
*Ss18*	1.06	1.40	1.63
*Rab34*	1.02	1.42	1.62
*BC026590*	0.96	1.47	1.60

**Table 3:** Top 20 genes synergistically repressed by T_3_ + CORT.

SYMBOL	T_3_ Fold Change	CORT Fold Change	T_3_ + CORT Fold Change
*Angptl4*	1.15	0.72	0.48
*Prl2c4*	0.99	0.83	0.55
*Zfhx3*	1.07	0.79	0.55
*Timp3*	1.06	0.75	0.56
*9430052C07Rik*	0.79	0.82	0.56
*Prl2c3*	0.97	0.77	0.57
*Tceal1*	0.83	0.76	0.58
*Actb*	0.93	0.63	0.58
*LOC671878*	0.96	0.70	0.60
*LOC100046616*	0.96	0.95	0.60
*Aqp5*	0.92	1.00	0.60
*Lbh*	1.00	0.83	0.61
*Tmsb4x*	0.99	0.69	0.61
*4933411D12Rik*	1.01	0.72	0.61
*Sox9*	0.95	0.89	0.61
*Tceal1*	0.88	0.70	0.61
*Plat*	1.13	0.74	0.62
*Prl2c2*	1.00	0.91	0.62
*Tomm6*	0.96	0.67	0.62
*Accn2*	1.10	0.74	0.62

### *In silico* analysis of open chromatin marks and transcriptional regulator binding sites

To identify candidate hormone-responsive enhancer elements, and to gain insight into possible transcriptional mechanisms behind the T_3_ + CORT- driven synergistic regulation of genes that we identified, publicly available ChIP-seq and ChAP-seq datasets [57–59], and the BEDOPS tool [61] were used to map previously identified GR and TR binding sites in the flanking regions of the synergistically regulated transcripts. Of the 153 synergistically regulated transcripts, genomic coordinates were retrieved for 110 genes (53 synergistically induced and 57 synergistically repressed genes) on the Mm10 assembly of the mouse genome. The other 43 transcripts for which no genomic coordinates could be retrieved were composed of cDNA clones with no or ambiguous genomic annotation.

**Fig 3:**
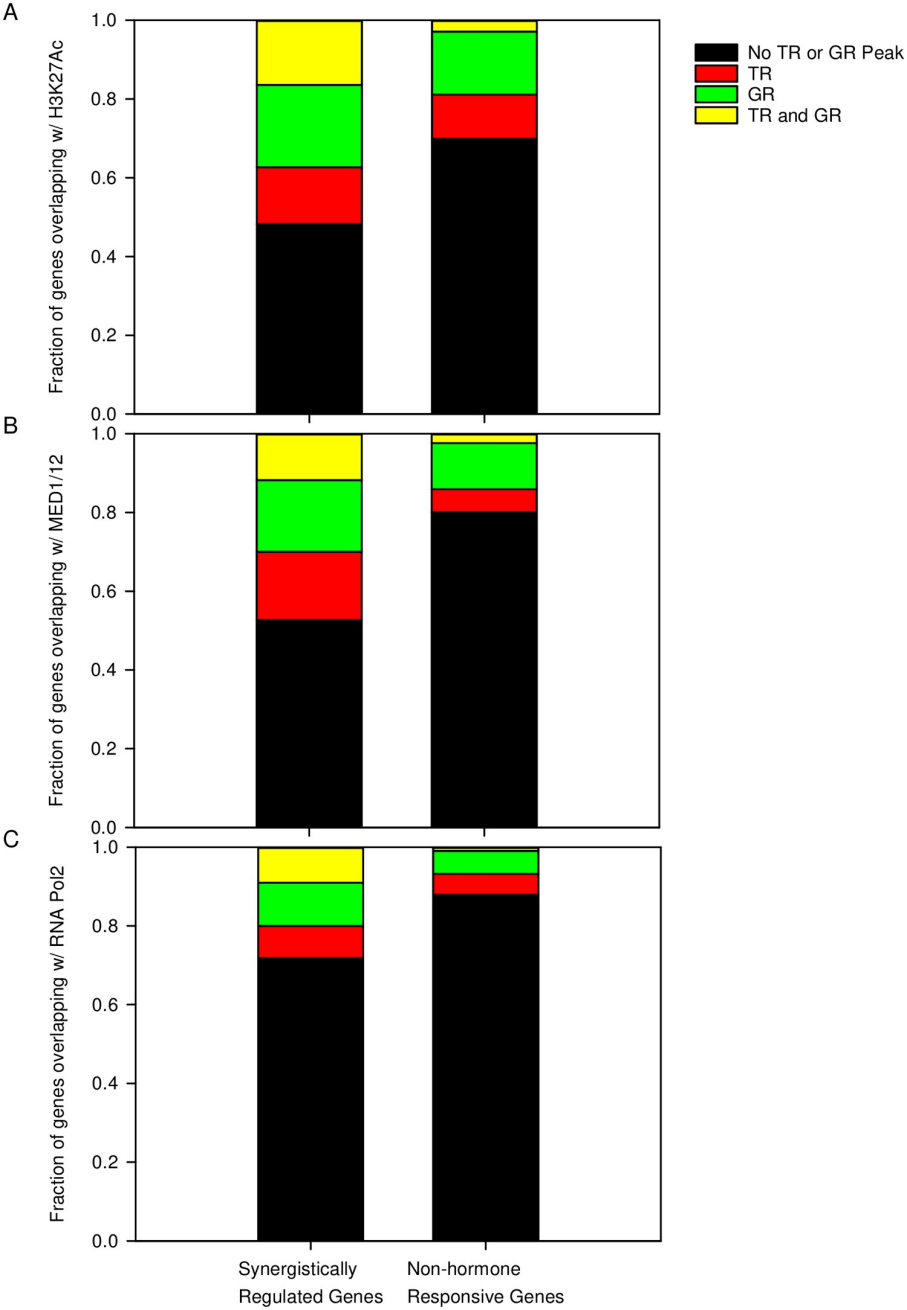
*In silico* analysis of TR and GR binding sites in the synergistically regulated and random, non-hormone regulated genes. Stacked bar charts showing the fraction of genes where TR and GR peaks could be mapped within 1 kb of open chromatin marked by **(A)** H3K27Ac peaks, **(B)** components of the Mediator subunits MED1 and MED12, and **(C)** RNA Pol2 are shown.

The search parameters for mapping analysis on the synergistically regulated genes were set to include the genomic region 50 kb upstream of the transcription start site (TSS) and 50 kb downstream of the transcription termination site (TTS), as well as the gene body itself [24]. Since the presence of a GR or TR peak proximal to open chromatin marks (H3K27Ac) is characteristic of distal regulatory regions [77, 78], we mapped GR and TR peaks within 1 kb of open chromatin as marked by H3K27Ac peaks [60]. Of the 110 synergistically regulated genes, 51.82% (57 out of 110) contained at least one GR or TR peak within 1 kb of an H3K27Ac peak (Fig 3A). Of the 51.82% of synergy genes, 20.90% contained only GR peaks, 14.54% contained only TR peaks, and 16.36% contained both a GR and a TR peak (S7 Table) mapping within 1 kb of the H3K27Ac mark. The presence of a GR or TR peak proximal to MED1 or MED12 peaks indicates a possible chromosomal looping interaction between distal regulatory regions or extragenic regions and the promoter of target genes [79, 80]. *In silico* analysis identified 47.27% (52 out of 110) of synergistically regulated genes (S8 Table) that contained a GR or TR peak within 1 kb of a MED1 or MED12 peak (Fig 3B). Of these 47.27% genes, 18.18% contained only GR peaks, 17.27% contained only TR peaks, and 11.82% contained both a GR and a TR peak. For candidate transcribed, hormone-responsive enhancer elements marked by RNA Pol2 peak [27], we found 28.18% (31 out of 110) of synergistically regulated genes (S9 Table) that contained a GR or TR peak within 1 kb of a Pol2 peak (Fig 3C). Of these 28.18% of synergy genes, 10.91% contained only GR peaks, 8.18% contained only TR peaks, and 9.09% contained both a GR and TR peak.

As a negative control, the same analysis was done on a random set of 300 transcripts/probe sets which were not called out as differentially expressed in any of the hormone treatments. We retrieved genomic coordinates for 206 out of the 300 transcripts on the Mm 10 assembly. Among the 206 transcripts, 30.1% of the genes (62 out of 206) had at least one TR or GR peak overlapping with an H3K27Ac peak (Fig 3A), 19.9% (41 out of 206) with at least one TR or GR peak overlapping with MED1 or MED12 peaks (Fig 3B), and 12.14% (25 out of 206) with at least one TR or GR peak overlapping with RNA Pol2 (Fig 3C).

Taken together, we found 4 synergistically regulated genes (*Klf9*, *Kdm6b*, *Slc3a2*, *Tob2)* where a GR and TR peak was mapped within 1kb of open chromatin marks (H3K27Ac), MED1/12 peaks, and RNA Pol2 peaks (S2 Fig). For the *Kdm6b* gene, GR and TR peaks mapped ~5kb upstream of the *Kdm6b* TTS, downstream of the flanking *Dnah2* gene which is not hormone regulated (S2A Fig). For the *Slc3a2* gene, the TR and GR peaks mapped ~3.75 kb downstream of TTS with additional GR peaks mapped within introns (S2B Fig). For the *Tob2* gene, TR and GR peaks mapped to the first exon of the gene (S2C Fig). To investigate the effect of T_3_ on the regulation of CORT responsive genes, we also conducted a similar analysis for the 33 genes where CORT-dependent gene regulation was enhanced or suppressed by T_3_ (Table 1 E3-6; S10–S12 Tables). Among the 33 genes, there were 28 that mapped to genomic coordinates on the Mm10 assembly. Genomic coordinates of representative genes, *Irak2*, *Lrp8*, and *Ccdc134*, with TR or GR peaks mapping within 1 kb of open chromatin marks are shown in S3 Fig. For the *Irak2* gene (S3A Fig), which exhibited CORT- dependent repression reversed by T_3_, TR peaks overlapping with H3K27Ac peak were mapped within introns, and a GR peak overlapping H3K27Ac peak was mapped in the first exon. For the *Lrp8* gene (S3B Fig) that exhibited CORT- dependent decrease in expression that was reversed by T_3_, a TR peak and overlapping H3K27Ac peaks mapped within an intron, and a distal GR peak overlapping with open chromatin marks mapped ~48.5kb downstream of the TTS, within the coding region of the neighboring *Cpt2* gene which is not hormone-regulated. For the *Ccdc134* gene whose CORT-dependent induction is suppressed by T_3_ (S3C Fig), TR peaks were found that mapped within an intronic region, and ~7.5 kb downstream of the TTS overlapping with open chromatin marks, MED1/12 peaks and RNA Pol2 peaks. A GR peak overlapping MED1/12 peaks was mapped ~20 kb upstream of the TSS within an exon of a neighboring gene *Mei1* which is not hormone-regulated.

Similar analysis was done for the 170 CORT-regulated genes that lose their CORT response in the presence of T_3_ (Table 1 B1-B2; S13–S15 Tables). We retrieved genomic coordinates for 129 of the 170 transcripts on the Mm 10 assembly. There were three CORT-regulated genes, *Adamtsl4*, *Samd4b*, *Sdc4*, where we could map both a GR and TR peak within 1 kb of open chromatin marks (H3K27Ac), MED1/12 peaks, and RNA Pol2 peaks (S4 Fig). For the *Adamtsl4* gene (S4A Fig), three GR peaks were mapped downstream of the TTS and a non-overlapping TR peak within an intron near the 3’ end of the gene. The GR peak located ~16 kb downstream of the *Adamts14* TTS overlaps with open chromatin marks, MED1/12 and RNA Pol2 peaks and the flanking *Mcl* gene which is not hormone-regulated. For the *Samd4b* gene (S4B Fig), two GR peaks were mapped ~7kb and ~21kb downstream of the TTS, with both GR peaks overlapping with all the chromatin marks and binding proteins analyzed. A TR peak ~46 kb downstream of the *Samd4b* TTS was also mapped, overlapping with all transcriptional regulators analyzed. All the flanking genes *Paf1*, *Med29*, *Zfp36*, *Plekhg2*, and *Rps16* are not hormone-regulated. For the *Sdc4* gene (S4C Fig), GR peaks overlapping with MED1/12 and RNA Pol2 peaks, and a TR peak overlapping with MED1/12 and H2K27Ac peaks were mapped within intronic and exonic regions of the gene body. The flanking genes *Rbpjl* and *Sys1* are not hormone-regulated.

### Functional annotation clustering

The DAVID tool v6.8 [69, 70] was used for functional annotation clustering of genes that were regulated by CORT alone, or synergistically regulated by T_3_ + CORT treatment. Because there were only 9 genes regulated by T_3_, we focused our functional annotation analysis on genes that were regulated by CORT alone (Fig 1A, section b; Table 1, B1-B2; Table E in S1 File), and genes that were synergistically regulated by T_3_ + CORT (Tables 2 and 3, Table K in S1 File). The top hits for CORT alone regulated genes (Table 4; Table N in S1 File) were functional annotation clusters for extracellular matrix, glutathione metabolism, transcription factor binding and regulation, and cell-cell adhesion. Genes that were synergistically regulated by T_3_ + CORT showed functional annotation clusters (Table 5; Table O in S1 File) for processes such as cell-cell adhesion, ubiquitin proteasome pathway, transcription factor binding and regulation, immune system responses, somatotropin hormone, and circadian rhythm.

**Table 4:** Functional annotation clustering of genes regulated by CORT alone.

Clusters	Enrichment Score
Extracellular matrix	1.896
Glutathione metabolism	1.587
Transcription factor binding	1.316
Cell adhesion	1.298
DEAD box helicase	1.225
Transcriptional regulation	1.183
Protein phosphorylation	1.179
Extracellular glycosylation	1.169

**Table 5:** Functional annotation clustering of genes synergistically regulated by T_3_ + CORT.

Clusters	Enrichment Score
Cell-cell adhesion	2.622
Ubiquitin ligase activity	2.425
Transcription factor binding	1.459
Innate immune response	1.459
Proteasome complex	1.454
Somatotropin hormone	1.285
Tetratricopeptide-like repeat motif	0.967
Mitcochondrial outer membrane	0.911
Negative regulation of transcription	0.872
Circadian regulation of gene expression	0.848

We also performed functional enrichment analysis for the 33 genes whose CORT-dependent response was altered in the presence of T_3_ (Table 1 E3-6; Table J and L in S1 File). We found functional annotation clusters for processes such as chemotaxis, extracellular localization, and transcription factor binding (Table 6; Table P in S1 File).

**Table 6:** Functional annotation clustering of genes where CORT-dependent response is altered in the presence of T_3_.

Clusters	Enrichment Score
Chemotaxis	1.71
Extracellular localization	1.17
Transcription factor binding	0.72
Cell membrane	0.23

### Validation of differential gene expression by RTqPCR

**Fig 4:**
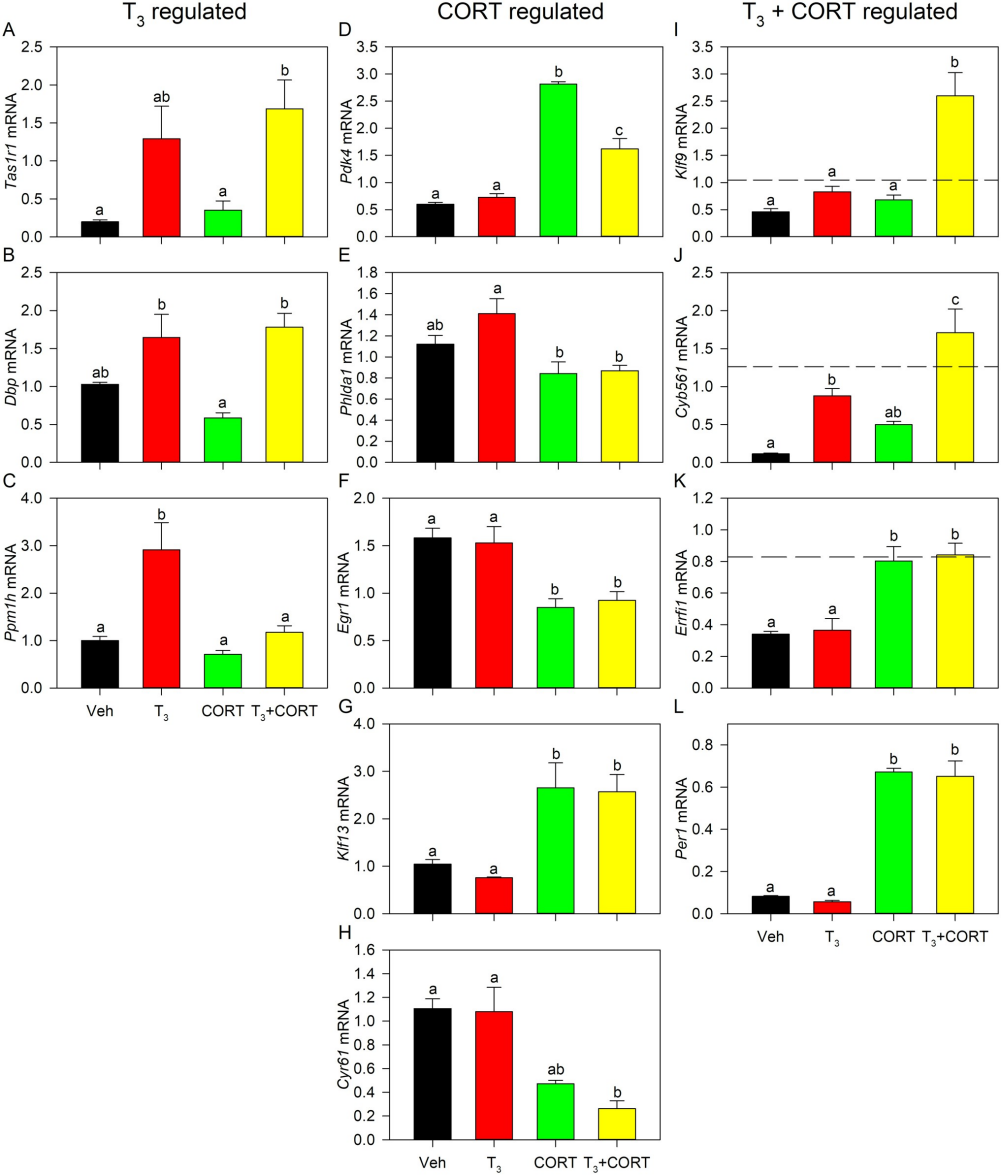
Quantitative RT-PCR validation of select target genes from microarray dataset. Gene expression analysis results are shown for *Tas1r1*, *Dbp*, and *Ppm1h* which were found to be T_3_-regulated **(A-C)**, *Pdk4*, *Phlda1*, *Egr1*, *Klf13*, and *Cyr61* which were found to be CORT-regulated **(D-H)**, and *Klf9*, *Cyb561*, *Errfi1*, and *Per1* which were found to be T_3_ + CORT regulated in the microarray dataset **(I-L)**. Bars represent the mean ± SEM, and letters above the means indicate significant differences among treatments (means with the same letter are not significantly different; Tukey’s multiple comparison test; *P* < 0.05). Dashed lines indicate the calculated additive effect of combined hormone treatment.

Validation of the microarray gene expression data sets was performed by analyzing mRNAs for genes which exhibited the strongest hormone response and distributed among the three hormone treatment groups (Fig 4). For genes called as differentially regulated by T_3_ (*Tas1r1*, *Dbp*, and *Ppm1h*) (Fig 4A–4C), gene expression analysis by RTqPCR showed statistically significant induction of *Tas1r1* in T_3_ + CORT treatment and a pattern of increased expression with T_3_ treatment (P = 0.0867). A pattern of induction of *Dbp* mRNA was observed in both T_3_ (*P* = 0.1278) and T_3_ + CORT (*P* = 0.0529) treatments but were not significantly different from vehicle, while a significant increase in expression of *Ppm1h* was observed for T_3_ treatment. For genes called as differentially regulated by CORT (*Pdk4*, *Phlda1*, *Egr1*, *Klf13*, and *Cyr61*) (Fig 4D–4H), induction of *Pdk4* and *Klf13* was observed in both CORT and T_3_ + CORT treatments while repression was observed for *Egr1* in both CORT and T_3_ + CORT treatments. A pattern of decrease in expression was observed for *Phlda1* (Veh vs. CORT: *P* = 0.2861, Veh vs. T_3_ + CORT: *P* = 0.3009) and *Cyr61* (Veh vs. CORT: *P* = 0.1050, Veh vs. T_3_ + CORT: *P* = 0.0284) in CORT and T_3_ + CORT but were not statistically significant. For genes called as differentially regulated by T_3_ + CORT (*Klf9*, *Cyb561*, *Errfi1*, and *Per1*) (Fig 4I–4L), RTqPCR validation showed induction of *Cyb561* in T_3_ and T_3_ + CORT, and induction of *Errfi1* and *Per1* in both CORT and T_3_ + CORT treatments, while an increase in expression of *Klf9* was only observed with T_3_ + CORT treatment. Upon further analysis, both *Cyb561* and *Klf9* were also shown to be synergistically regulated by combined hormone treatment. The comparison of fold change in gene expression as determined by microarray vs RT-qPCR is detailed in S16 Table. These results simply mean that the magnitude change in gene expression discovered by microarray, when comparing a hormone treatment to the control, was confirmed by targeted RTqPCR analysis for many, but not all genes tested.

### Identification of a putative enhancer element in the *Cyb561* gene

Although the *in silico* analysis of mouse ChIP-seq data did not show co-localization of all transcriptional regulators (TR, GR, H3K27Ac, MED1/12, and RNA Pol2) mapping within 1 kb of each other to the *Cyb561* locus, identification of an enhancer was focused on the *Cyb561* gene since it showed the strongest synergistic induction with T_3_ + CORT treatment. In addition, *Cyb561* is included in the list of genes where we could map a TR or GR peak within 1 kb of H3K27Ac or MED1/12 peak (S17 Table). We identified a candidate enhancer using *in silico* analysis from human data as the bulk of publicly available data is in the human genome assembly. The region we identified had characteristics of genomic enhancer elements such as marks for an open chromatin structure and was conserved between vertebrate species [77, 78, 81]. Data from the University of California Santa Cruz (UCSC) Genome Browser and ENCODE were used to conduct *in silico* analysis to identify a putative hormone-responsive enhancer module in the human *Cyb561* gene (*hCyb561*). A genomic region located ~15.5 kb upstream of the *hCyb561* TSS was chosen for matching the following criteria: contains a GR binding peak, is a transcription factor binding hotspot, has marks of an open chromatin environment such as H3K27 hyperacetylation and DNase I hypersensitivity, and was conserved among vertebrates (S5A Fig). This candidate enhancer initially identified in the *hCyb561* gene has a homologous region in *mCyb561* gene located ~9.0 kb upstream of the *mCyb561* TSS that overlapped with a TR peak, and with RNA Pol2 peaks and MED1/12 peaks mapping to flanking regions (S5B Fig). A TR peak also mapped to intron 3 of the *mCyb561* gene body. These chromatin marks, and transcription factor and RNA Pol2 binding peaks are suggestive of regions in the genome that may play important functional roles in transcriptional regulation.

**Fig 5:**
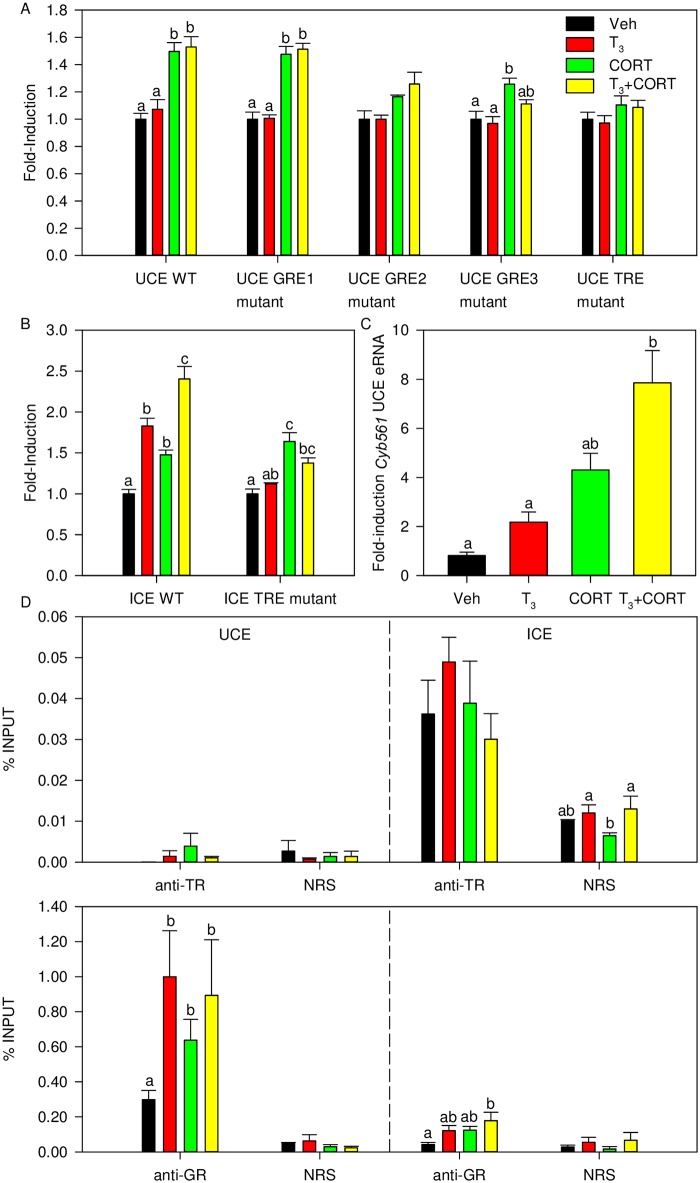
Identification of putative upstream enhancer and intronic enhancer involved in synergistic transcriptional regulation of the *Cyb561* gene. Hormone-dependent transactivation of **(A)** the UCE and GRE1, GRE2, GRE3, and TRE1 mutant constructs, and **(B)** the ICE and TRE mutant constructs through dual luciferase assay after treatment with either Veh, T_3_, CORT, or T_3_ + CORT. (**C**) Hormone treatment induces *Cyb561* eRNA in HT-22 cells. **(D)** TR constitutively associates at the ICE while GR association is higher at the UCE where hormone-dependent GR association is observed. Enhancer-reporter assays were repeated at least 3 times with consistent results. Bars represent the mean ± SEM in fold-induction for enhancer reporter assays, or % input for ChIP analysis, and letters above the means indicate significant differences among treatments (means with the same letter are not significantly different; Tukey’s multiple comparison test; *P* < 0.05).

The two *mCyb561* candidate enhancer regions, an 850 bp fragment which we designated the upstream *Cyb561* enhancer (UCE; -9900 to -9051 bp from TSS) and 410 bp fragment in intron 3 which we designated the intronic *Cyb561* enhancer (ICE; +7567 to +7977 bp from TSS), were cloned into pGL4.23 for use in enhancer-reporter assays (Fig 5A). The UCE contains 3 putative GREs which were designated as GRE1 (-9868 to -9854 bp from TSS), GRE2 (-9589 to -9575 bp from TSS), and GRE3 (-9439 to -9425 bp from TSS) and one putative TRE designated as TRE1 (-9243 to -9228 bp from TSS) (S5C Fig). Among the GREs in the UCE, GRE2 showed the highest score in the LASAGNA search algorithm (score = 16.21; Table Q in S1 File). The ICE contains 3 putative GREs which were designated GRE1 (+7605 to +7619 bp from TSS), GRE2 (+7658 to +7673 bp from TSS), and GRE3 (+7728 to +7742 from TSS) and 1 putative TRE which was designated as TRE1 (+7832 to +7847 bp from TSS) (S5C Fig). Among the GREs in the ICE, GRE1 showed the highest score in the LASAGNA search algorithm (score = 13.37; Table R in S1 File). The UCE supported CORT but not T_3_-dependent transactivation and did not show synergistic activity with combined hormone treatment (Fig 5A). Mutation of GRE2 of the UCE led to the loss of CORT-dependent transactivation (Fig 5A) while mutation of GRE3 led to decreased CORT-transactivation. Mutation of the TRE also led to a loss of hormone-dependent transactivation in the UCE. Lastly, we found that the ICE supported both T_3_-dependent and CORT-dependent transactivation but did not show synergistic activity with combined hormone treatment (Fig 5B). Mutation of the TRE in the ICE led to a loss of T_3_-dependent activity.

Data from Kagey et al. (2010) showed that the putative UCE was associated with RNA Pol2 and Mediator ChIP-seq peaks (S5B Fig), which is indicative of enhancer RNA (eRNA) transcription. To determine if the *mCyb561* UCE is transcribed into eRNA, transcripts from the UCE were measured by RTqPCR and we found that an eRNA transcribed from *mCyb561* UCE was also synergistically induced by combined hormone treatment (Fig 5C) similar to the pattern of hormone regulation of the *Cyb561* mRNA (Fig 4J).

To determine if GR and TR are recruited to chromatin at the regions of the *Cyb561* UCE and ICE, we conducted ChIP assays using antiserums to TR or GR. We saw no significant TR ChIP signal at the UCE. By contrast, at the ICE the TR ChIP signal was significantly higher than the NRS control, but the signal was not affected by hormone treatment, consistent with TRs being constitutively resident in chromatin (Fig 5D). The GR ChIP assay showed hormone-dependent recruitment of the GR to chromatin at the UCE and ICE, and that GR ChIP signal was higher at the UCE compared with the ICE (Fig 5D). We did not observe GR ChIP signal at 5 kb upstream of the *Cyb561* TSS or at an intronic region located 5 kb downstream of the TSS (S7 Fig).

## Discussion

Thyroid hormones and GCs have been shown to have significant roles in regulating basic neuronal developmental processes, especially in the hippocampus. Thyroid hormone regulates key processes in early brain development such as neurogenesis, neuronal migration and differentiation, myelination, and synaptogenesis [82–86]. Maternal hypothyroidism has been shown to lead to abnormal brain development [87] and a growing body of evidence demonstrate that even moderate disturbances in maternal thyroid function during fetal gestation can influence neuronal programming and increase the risk of developing neurodevelopmental disorders long after birth [84]. Similarly, the role of GCs in regulating the stress response, and its consequent effects on neuronal development are also well established [88–90]. Early life stress leads to reprogramming of the hypothalamic-pituitary-adrenal (HPA) axis that is associated with increased risk for neuropsychiatric disorders later on in life [91]. Effect of GCs on the hippocampus varies with length of exposure with acute stress stimuli enhancing memory consolidation [13, 14] while chronic stress promotes neuronal atrophy that leads to impaired performance in learning and memory related tasks [8, 9, 12].

In mammals, TH and GCs act in synergy to promote both lung and gut development in rat models [92, 93]. The synergistic role of TH and GC in development is best exemplified in amphibians, where TH increases the sensitivity of tadpole tissues to GCs thereby accelerating metamorphosis [56]. A study by Kulkarni and Buchholz (39) used a microarray platform to survey the effect of GC on large-scale gene expression changes during TH-driven tadpole tail resorption. Here we report results to a similar approach to surveying TH and GC synergy in a mouse hippocampal neuronal model.

### Genes regulated by T_3_

Since there were only 9 genes regulated by T_3_ based on the FC cut-off criteria, and only 3 of those genes were exclusively regulated by T_3_, functional annotation analysis was not possible. These results suggest that although HT22 cells express multiple isoforms of TRα and TRβ [49], the differentiated mouse hippocampal neurons may be largely unresponsive to T_3_ as we only found 9 T_3_- responsive genes, and only three (*Ppm1h*, *Npr3*, and *Cdon*) of those were exclusively regulated by T_3_. The *Ppm1h* (*Nerpp-2c*) gene encodes a protein phosphatase expressed in rodent brain and neuronal cell lines, and functions in regulating neurite growth [94]. The *Npr3* gene encodes a natriuretic peptide receptor that has a minor effect in disrupting the axonal branching of neurons from the dorsal root [95] while the *Cdon* gene encodes a cell surface protein which has been shown to participate in oligodendrocyte differentiation and myelination [96]. Based on the genes exclusively regulated by T_3_, it seems that a major function of T_3_ in our cell culture system is to regulate processes involved in axonal branching.

### Genes regulated by CORT

There was a larger number of genes differentially regulated by CORT as compared to T_3_. Functional annotation analysis on the subset of genes which were regulated by CORT alone and for which CORT response was lost in the presence of T_3_ revealed terms related to well-established processes related to neuronal morphology and migration, such as extracellular matrix (ECM), extracellular glycosylation, and cell adhesion. Some of the genes we found uniquely clustered under CORT-responsive cell adhesion were *Nuak1* which regulates terminal axon branching via capture and immobilization of mitochondria at nascent presynaptic sites in cortical neurons [97], and *Vcan* which encodes an ECM protein that also facilitates cell-adhesion and mediates some signaling mechanisms in the peripheral nervous system [98]. The ECM-related gene *Ccn1* (also known as *Cyr61*) has been shown to be essential for dendritic arborization in hippocampal neurons [99]. Among all the genes clustered in the ECM category, only the *Col3a1* gene, encoding collagen type III alpha, has been previously shown to be CORT regulated [100]. Extracellular matrix proteins play important functions in the maintenance of a microenvironment for neuronal migration, axonal growth, and synaptogenesis, [101–104], and ECM removal permits modifications in neuronal architecture that may enhance synaptic plasticity [105].

Interestingly, we also found enrichment for terms related to glutathione metabolism. While the mechanisms for antioxidant defense in the brain are not completely understood, there is evidence that brain glutathione concentrations are linked to neuronal processes such as learning and memory, and neurodegeneration and aging [106–108]. Recent studies showed that synaptic NMDA receptor activity is actually coupled to control of the glutathione system such that antioxidant defense mechanisms in active neurons are able to match their highly active state to protect against neurodegeneration [109]. These findings support the known outcomes of the stress response in the brain, and more importantly, identify new GC responsive genes that may help explain the established effects of GCs in memory consolidation and reconsolidation.

### Genes synergistically regulated by T_3_ and CORT

Genes that are synergistically regulated by T_3_ and CORT showed unique enrichment terms for ubiquitin ligase activity, innate immune response, proteasome complex, somatotropin hormone, and circadian rhythm regulation. The circadian rhythm is controlled in the suprachiasmatic nucleus in the hypothalamic region of the brain by a set of oscillating transcription factors [110–112]. Glucocorticoid concentrations are controlled in an oscillatory fashion by the circadian rhythm and at the same time, GCs also adjust peripheral circadian clocks [113, 114]. Consistent with our results linking hormone response to circadian rhythm, previous evidence indicate that there is an enrichment of *Klf9* binding in genes involved in the control of circadian rhythm [115], and that *Klf9* is also expressed in a circadian manner in human epidermis [116]. We previously identified *Klf9* as one of the most strongly synergistically regulated genes by T_3_ and CORT which suggests that the synergistic effect of T_3_ and CORT on expression of circadian rhythm genes may be indirectly attributed to the direct, strong synergistic induction of *Klf9* expression by T_3_ and CORT.

There was also an enrichment for terms related to transcription factor binding which included genes such as *Klf9* and *Sox9*, as well as genes related to chromatin remodeling and long-range chromatin interactions such as *Smarcd2* and *Med16*. This is in accordance with the expected role of the TR and GR as ligand-mediated transcription factors [18, 22, 117]. Many of the immediate response genes regulated by TH and GCs are also transcription factors themselves [118, 119] supporting the hypothesis that TH and GCs act synergistically to orchestrate large scale gene regulatory cascades subsequently resulting in accelerated neuronal development or enhanced response to hormone induction.

We also found enrichment for terms related to somatotropin growth hormone, which include genes *Prl2c2*, *Prl2c3*, and *Prl2c4*. Thyroid hormone and GC synergy in regulating growth hormone receptor expression in rodent pituitary cells has actually been reported as early as the 1970s [120] and is one of the well-established processes regulated by both hormone axes [121, 122]. While our results are consistent with the known effect of TH and GC in the hypothalamus and pituitary cells which then consequently affects the timing of development and regulation of body size, prolactin also has specific functions in neurodevelopment and neuroprotection in the hippocampus [123, 124]. For example, prolactin administration exerts a neuroprotective effect on hippocampal neurons in both *in vivo* and *in vitro* rodent models of excitotoxicity [125, 126]. Prolactin also protects neurogenesis in the dentate gyrus in a rodent model of chronic stress [126]. There is evidence for extrahypothalamic expression of prolactin genes and the prolactin receptor in many brain regions including the hippocampus [123, 127] which points to the likely existence of a prolactin system in the hippocampus independent of the hypothalamus. This synergistic regulation of the prolactin genes by TH and GC broadens our understanding of the pleiotropic effects of the growth hormones in the brain, and adds new dimension to the regulation of neuronal processes by the two hormone axes.

Enrichment for terms related to ubiquitin ligase activity and immune response are consistent with emerging evidence showing that these two processes may actually have profound effects on neuronal processes such as learning and memory. For example, several evidence implicate activity-dependent protein degradation as one of the processes important in contextual memory formation [128–130]. In addition, inhibition of the proteasomal pathway in a murine model resulted in impaired performance in spatial learning tasks which may be associated with the increase in β-amyloid protein aggregates [131]. Ubiquitination also controls the trafficking and recycling of glutamate receptors which is an important process affecting synaptic plasticity [131]. Few studies have looked into the role of TH or GC regulation of proteasomal pathway genes. A study by Wuwongse, Cheng (132) investigated the involvement of ubiquitin-proteasome pathway in the disease progression of a CORT and amyloid-beta induced Alzheimer’s disease model in primary hippocampal neuron culture. However, to our knowledge, no other study has shown synergistic TH and GC regulation of the ubiquitin proteasome pathway in hippocampal neurons.

Similarly, there is evidence for the important role of neuroinflammation in influencing learning and memory related neuronal processes [133–135]. Neuroinflammation has been associated with impaired learning and memory in rodent models possibly due to damage of hippocampal neuronal cells [136, 137]. There is also evidence for long-lasting decreases in dendritic spine density as a result of transient immune response in a murine model [138] highlighting the strong influence of neuroinflammatory processes on neuronal morphology and consequently learning and memory.

Our functional annotation analysis results suggest that T_3_ and CORT likely coordinate many neuronal processes to accelerate neuronal development by enhancing hormone response through the synergistic induction of gene regulatory networks. These results are consistent with previous evidence showing T_3_ and CORT synergistic regulation of tadpole tail morphogenesis, and gut and lung maturation in rodent models [56, 92, 93]. More importantly, our data suggests that T_3_ and CORT affects learning and memory through neuroprotective effects of prolactin genes, modulation of the proteasome–dependent protein turn-over important for long term potentiation, and regulation of neuroinflammatory responses.

### *In silico* analysis provide mechanistic insights into synergistic regulation

HT-22 cells showed very few genes responsive to T_3_ treatment alone and that a majority of the genes in our microarray dataset were CORT-responsive. However, we found that for 10 genes T_3_ treatment seemed to enhance CORT-responsiveness while 147 genes showed differential expression only in the presence of T_3_ + CORT. This is in line with the proposed function of ligand-bound TR as a transcriptional switch that opens up the chromatin environment to facilitate the binding of transcriptional co-regulators and transcription machinery [49, 117, 139, 140]. This dual function of TR, as a transcriptional repressor in the absence of a ligand or transcriptional activator in the presence of a ligand, plays an important role in regulating transcription of genes required for mammalian developmental transitions [24, 141–143]. Conversely, altered CORT response in the presence of T_3_ may occur through facilitated loading of TR only when GR is bound at target sites. Binding of GR can alter the local chromatin environment to allow the recruitment of other transcription factors [144, 145]. This phenomenon of GR-assisted loading has been demonstrated in the crosstalk between GR and the estrogen receptor [145, 146]. It is possible that the mechanism behind synergistic gene regulation by TH and GC is not unique to the *Klf9* gene [49] but is shared with other hormone-responsive genes, where the activation of multiple nuclear hormone receptors alters chromatin assembly and modulates the accessibility of hormone response elements to each receptor [146].

To further support the possible mechanisms for transcriptional regulation by these hormone axes, we mapped potential GR and TR binding sites to the surrounding genomic regions of the synergistically regulated genes. To strengthen the analysis, we only mapped GR or TR peaks which were within 1kb of a H3K27Ac peak, MED1/MED12 peaks, and RNA Pol2 peaks, as these marks are indicative of distal regulatory regions [77, 78, 81]. Among the 110 synergistically regulated genes which we could map to genomic coordinates in the Mm10 assembly, there were 57 genes (51.82%) that contained a GR or TR peak within 1 kb of a H3K27Ac peak and within 50kb upstream of the TSS and 50kb downstream of the TTS. The other 53 genes for which we could not map these GR or TR peaks may be regulated by a secondary transcription factor that is induced by T_3_ and CORT treatment, but this needs to be experimentally validated. There were also 52 genes (47.27%) which contained a GR or TR peak within 1 kb of a MED1/MED12 peak. The close localization of these GR and TR peaks to subunits of the Mediator complex suggest that some long-range chromosomal interaction may be involved in their hormonal regulation [27] but again, this needs to be experimentally validated. Lastly, we found 31 genes (28.18%) which contained a GR or TR peak within 1 kb of an RNA Pol2 peak. The close localization of these GR and TR peaks with the RNA Pol2 peaks in non-coding regions suggest that these regions may be transcribed into eRNAs [27], adding emphasis to the role of eRNAs in the regulation of hormone-dependent transcription. Overall, we identified 3 genes (*Kdm6b*, *Slc3a2*, *Tob2*) which were synergistically regulated by T_3_ + CORT similar to the *Klf9* gene [49] and contained GR and TR peaks mapping within 1 kb to open chromatin marks (H3K27Ac peaks), components of the Mediator complex (MED1/12), and RNA Pol2. These results further support our hypothesis that the molecular mechanism behind the synergistic action of TR and GR is not unique to the *Klf9* gene, and most likely occurs in other genes synergistically regulated by TH and GC.

We performed a similar analysis for TR and GR binding sites co-localized with open chromatin marks, MED1/12 and RNA Pol2 peaks for genes which were CORT-responsive but whose CORT-response was lost in the presence of T_3_ and for genes which were CORT-responsive but whose CORT-response was either enhanced or reduced in the presence of T_3_. We found that TR and GR peaks map at diverse sites similar to the results we obtained from mapping analysis for the synergistically regulated genes, indicating that the mechanisms of hormone-dependent regulation of all these genes with differing hormone-responses may occur through similar mechanisms. Although we were not able to map TR or GR peaks to all of the genes, it is possible that the hormone-responsive loci which act to regulate these genes may be located further than 50kb away from the gene, or that they are secondary target genes.

Lastly, when *in silico* transcription factor binding analysis was performed on a randomly selected set of genes which were not called as differentially expressed in response to any hormone treatment, the fraction of genes which contained TR or GR binding sites co-localized with open chromatin marks, MED1/12 and RNA Pol2 peaks was significantly less. This suggests that the co-localization of these TR and GR binding sites is likely not a random occurrence and supports our *in silico* analysis on the possible contribution of TR and GR in influencing chromatin structure to help explain synergistic regulation of target genes.

### Molecular basis for synergistic regulation of *Cyb561* gene

Since the microarray results and RTqPCR validation were mostly consistent, and because *Cyb561* was the most synergistically up-regulated gene, we focused our attention on deciphering its hormone-dependent regulatory mechanisms. The *Cyb561* gene encodes a transmembrane electron transport protein involved in the recycling of ascorbate, a cofactor in neuropeptide amidation in neurosecretory vesicles [147–149]. Neuropeptide amidation is an important step in their activation and knockdown of a *Cyb561* homologue in *Drosophila* results in impaired performance in learning and memory tasks concurrent with decreased amidation of neuropeptides [150, 151].

To determine if the synergistic regulation of the *Cyb561* gene by T_3_ and CORT occurs through similar molecular mechanisms as the *Klf9* gene [49], we first identified and cloned the UCE and ICE for testing in enhancer-luciferase assays. Both UCE and ICE contain candidate GREs and TREs which we mutated and tested for hormone-dependent transactivation. Results of the enhancer-reporter assay did not show synergistic transactivation of the UCE or ICE with T_3_ + CORT treatment. However, the UCE was CORT-responsive and the CORT-response was abolished by mutating GRE2. Mutation of the TRE in the UCE eliminated the CORT induction suggesting that TR binding is required for the CORT response. The ICE showed T_3_- and CORT-dependent transactivation. Mutating the TRE in the ICE abolished the T_3_ but not the CORT induction which implies that the crosstalk between TR and GR is not as salient in the ICE compared to the UCE.

Our ChIP analyses for TR and GR showed that TR was constitutively resident in chromatin at the ICE but not the UCE, while hormone-dependent recruitment of GR to chromatin was higher at the UCE compared to the ICE. These data, along with the localization of MED1/12 peaks [27] between the *Cyb561* promoter and UCE, and our preliminary findings of reduced basal expression of *Cyb561* with MED1 knockdown (S6 Fig) suggest that the synergistic regulation of *Cyb561* may be due to long-range chromosomal interaction [152], and perhaps cooperative *cis*-regulatory activity [153] between the UCE, promoter and/or ICE. This could explain why plasmid reporter assays using either the UCE or ICE did not show synergistic regulation by T_3_ + CORT, and more importantly, they emphasize the caveat that enhancer-reporter assays may not accurately reflect the chromatinized environment of the endogenous enhancers. Apart from MED1/12 peaks, RNA Pol2 binding sites [27] were also found flanking the UCE indicating it was transcribed as enhancer RNA. We measured transcription at the UCE in response to hormone treatment and found that the UCE was indeed transcribed and synergistically induced by T_3_ + CORT similar to the expression pattern of the *Cyb561* mRNA. We also have preliminary evidence for a novel eRNA associated with the *Per1* gene which was differentially expressed by T_3_ and CORT in the same pattern as *Per1* mRNA. This suggests that eRNA induction may be a common regulatory theme that may play a role in hormone-dependent transcriptional regulation in the brain.

## Conclusion

To our knowledge, our microarray dataset represents the first of its kind to identify a vast array of genes that are coordinately regulated by TH and GC in any cell type in mouse, and the first to investigate the synergistic effect of TH and GC which have long been known to independently affect learning and memory. Our dataset shows some overlap with a previous study examining the effect of TH, retinoic acid, and GC signaling on the expression of specific genes in primary mouse cerebrocortical cells [37]. A similar study using microarray analysis to identify TH and GC gene regulation patterns during development of *X*. *tropicalis* tadpoles also found a diverse set of gene regulation effects for T_3_, CORT, and T_3_ + CORT, and similarly identified the proteasomal pathway as a synergistically regulated process [39]. Our dataset also suggests that differentiated hippocampal neurons are more responsive to GCs, with TH mainly modulating the response to GCs. Our microarray analysis identified several well-established processes in learning and memory that are regulated by GCs such as cell adhesion, cytoskeletal remodeling, and extracellular matrix proteins implicated in the formation and maintenance of synapses, and axon guidance navigation. More importantly, we identified several genes apart from *Klf9* which were synergistically regulated by TH and GC and were shown to be associated with well-known functions of TH and GC in controlling transcription and circadian rhythm. Our findings also highlights the significance of neuroinflammation [133, 134], neuroprotective effect of prolactin genes [123–126], and proteasomal-dependent degradation [128–132, 154] pathways as processes relevant to how TH and GC may coordinately influence learning and memory. Taken together, our dataset are consistent with established hormone-regulated pathways, and identify novel hormone-induced genes and mechanisms by which T_3_ and GC may coordinately and synergistically regulate neurodevelopment, and learning and memory processes.

## Supporting information

S1 Fig Generalized expression patterns of genes synergistically regulated by T_3_ + CORT. Box plots were created to represent the different regulation patterns of synergistically regulated genes by T_3_ plus CORT. The heavy dashed lines represent the Log_2_ of 1.5 fold-change cut-off for differential expression.
(TIF)

S2 Fig Visualizations of the genomic coordinates of synergistically regulated genes with TR and GR peaks mapping to open chromatin marked by H3K27Ac peaks, the Mediator subunits Med1 and Med12, and RNA Pol2. Genomic plots showing **(A)** *Kdm6b*, **(B)** *Slc3a2*, **(C)** *Tob2*, were made using the Integrative Genomics Viewer tool [155, 156]. Regions highlighted in gray correspond to the gene body while those in green correspond to the genomic regions where the TRs and GRs can be found.
(TIF)

S3 Fig In silico analysis of TR and GR binding sites in CORT-responsive genes whose CORT-response is lost in the presence of T3. Genomic plots showing representative genes **(A)** *Irak2*, **(B)** *Lrp8*, **(C)** *Ccdc134* were made using the Integrative Genomics Viewer tool [155, 156]. Regions highlighted in gray correspond to the gene body while those in green correspond to the genomic regions where the TRs and GRs can be found.
(TIF)

S4 Fig In silico analysis of TR and GR binding sites in CORT-responsive genes whose CORT-response is either enhanced or reduced in the presence of T3. Genomic plots showing representative genes **(A)** *Adamtsl4*, **(B)** *Samd4b*, **(C)** *Sdc4* were made using the Integrative Genomics Viewer tool [155, 156]. Regions highlighted in gray correspond to the gene body while those in green correspond to the genomic regions where the TRs and GRs can be found.
(TIF)

S5 Fig Identification of putative upstream enhancer and intronic enhancer involved in synergistic transcriptional regulation of the Cyb561 gene. **(A)** Genomic plot of the human *Cyb561* locus showing characteristic open chromatin marks (H3K27 hyper acetylation, DNAse I sensitivity, transcription factor binding) and vertebrate conservation plotted using the UCSC Genome Browser [157] based on the hg19 build of the human genome. **(B)** Genomic plot of the mouse *Cyb561* locus and open chromatin marked by FAIRE-seq and H3K27Ac peaks, the Mediator complex subunits MED1 and MED12, GR, and TR peaks determined by ChIP-seq [27, 57–59]. Data were plotted using the Integrative Genomics Viewer [155, 156] on the mm10 build of the mouse genome. **(C)** Alignments of the WT UCE and ICE constructs with generated mutants. Conserved bases in the GREs are highlighted in green while conserved bases in the TREs are highlighted in red. Yellow highlights indicate bases which were edited in the respective mutants.
(TIF)

S6 Fig Knockdown of Mediator complex subunit Med1 in HT-22 cells results in reduced basal Cyb561 expression. HT22 cells were stably transduced with a *Med1* shRNA lentiviral construct or with a scrambled shRNA lentiviral control. Stably transduced cells were selected with 2 μg/μL puromycin. Cells were grown in selection media for three passages before harvest and RNA extraction. We measured gene expression by RTqPCR for **(A)** *β-actin*, **(B)** *Med1*, and **(C)** *Cyb561*. There was no significant effect of *Med1* knockdown on the expression of the reference gene *β-actin* used for normalization of RNA transcripts. HT-22 cells transduced with sh*Med1* exhibit significantly reduced *Med1* mRNA expression at 62.3% knockdown compared to the scrambled control. Basal *Cyb561* expression in HT-22 cells transduced with sh*Med1* is significantly reduced compared to scrambled control.
(TIF)

S7 Fig GR does not associate to -5.0 kb upstream of the TSS and intron 1 of Cyb561 gene. Neither the region -5.0 kb upstream of TSS nor +5.5 kb downstream of TSS (intron 1) of the *Cyb561* gene exhibit GR-association when tested through ChIP qPCR. Bars represent the mean ± SEM in % input for ChIP analysis, and letters above the means indicate significant differences among treatments (means with the same letter are not significantly different; Tukey’s multiple comparison test; *P* < 0.05).
(TIF)

S1 Table Primers used for RT-qPCR. (DOCX)

S2 Table Primers used for cloning and site-directed mutagenesis. (DOCX)

S3 Table Primers used for ChIP. (DOCX)

S4 Table Genes differentially regulated by T3. (DOCX)

S5 Table Top 10 genes induced and repressed by CORT. (DOCX)

S6 Table Top 10 genes induced and repressed by T3 + CORT. (DOCX)

S7 Table In silico analysis of synergistically regulated genes for GR and TR peak binding within 1kb of open chromatin marks (H3K27Ac). (DOCX)

S8 Table In silico analysis of synergistically regulated genes for GR and TR peak binding within 1kb of MED1/12 peaks. (DOCX)

S9 Table In silico analysis of synergistically regulated genes for GR and TR peak binding within 1kb of RNA Pol2 peaks. (DOCX)

S10 Table In silico analysis of genes whose CORT-response is altered by T3 for GR and TR peak binding within 1kb of open chromatin marks (H3K27Ac). (DOCX)

S11 Table In silico analysis of genes whose CORT-response is altered by T3 for GR and TR peak binding within 1kb of MED1/12 peaks. (DOCX)

S12 Table In silico analysis of genes whose CORT-response is altered by T3 for GR and TR peak binding within 1kb of RNA Pol2. (DOCX)

S13 Table In silico analysis of CORT-responsive genes that lose CORT response with T3 for GR and TR peak binding within 1kb open chromatin marks (H3K27Ac). (DOCX)

S14 Table In silico analysis of CORT-responsive genes that lose CORT response with T3 for GR and TR peak binding within 1kb of MED1/12 peaks. (DOCX)

S15 Table In silico analysis of CORT-responsive genes that lose CORT response with T3 for GR and TR peak binding within 1kb of RNA Pol2 peaks. (DOCX)

S16 Table Summary of differential expression analysis results for microarray and RT-qPCR validation. (DOCX)

S17 Table In silico analysis of synergistically regulated genes for GR or TR binding within 1 kb of open chromatin marks (H3K27Ac) or MED1/12 peaks. (DOCX)

S1 File Contains table B-R. (XLSX)
