# Supplementary figures and images for: Coordinated transcriptional regulation by thyroid hormone and glucocorticoid interaction in adult mouse hippocampus-derived neuronal cells
Source: PLoS One. 2019 Jul 26;14(7):e0220378. doi: 10.1371/journal.pone.0220378 (PMC6660079; doi:10.1371/journal.pone.0220378)

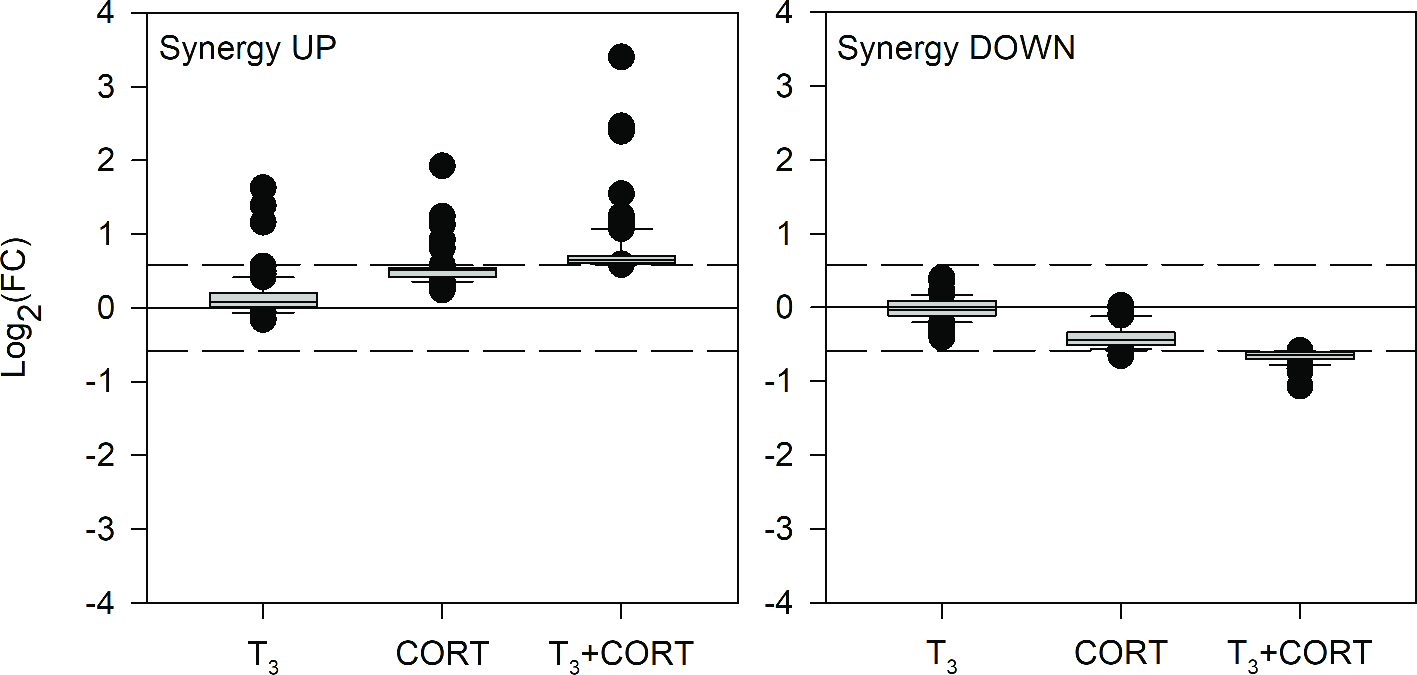

Supplement: S1 Fig — Box plots were created to represent the different regulation patterns of synergistically regulated genes by T3 plus CORT. The heavy dashed lines represent the Log2 of 1.5 fold-change cut-off for differential expression. (TIF) [file pone.0220378.s001.tif]

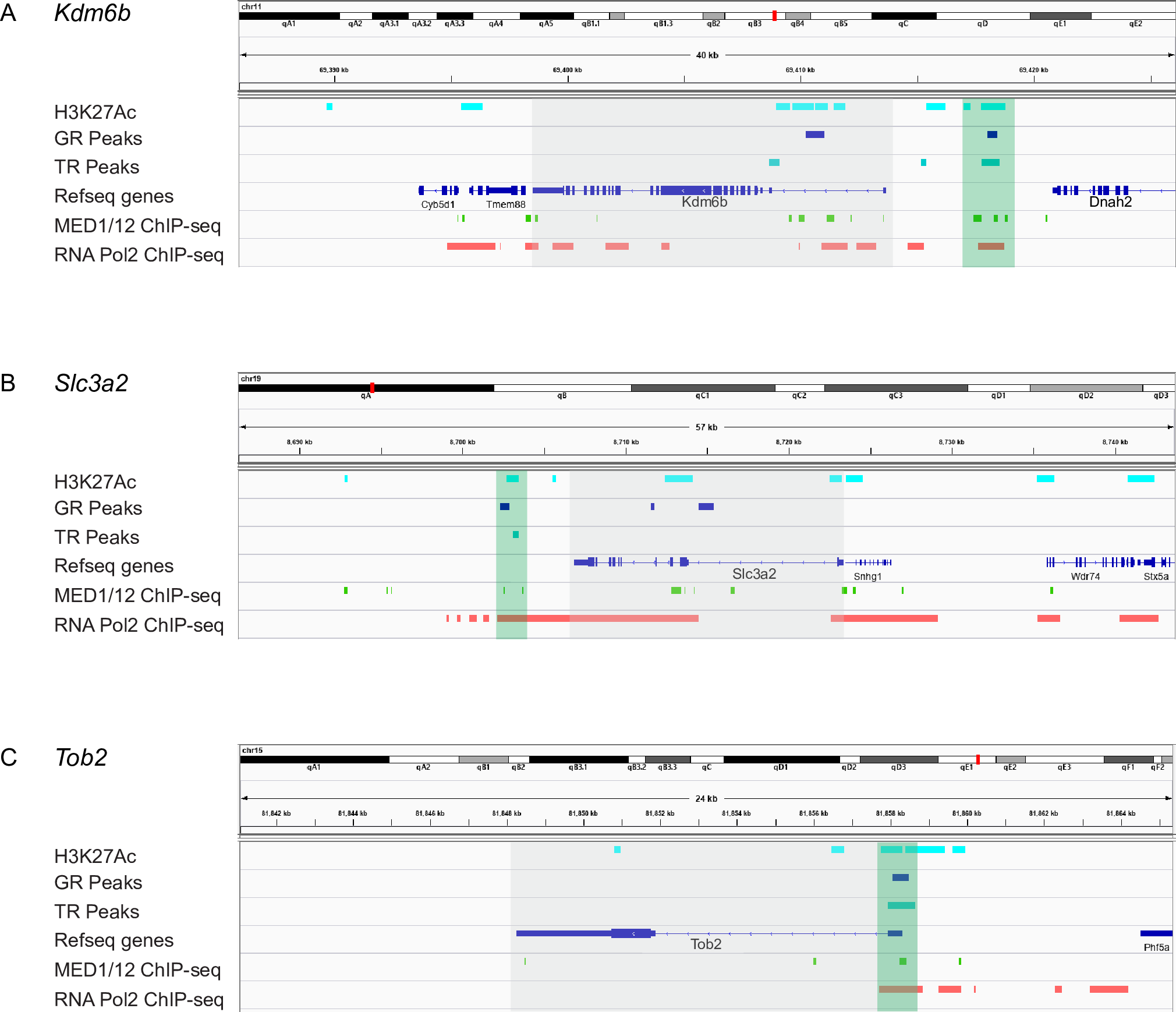

Supplement: S2 Fig — Genomic plots showing (A) Kdm6b, (B) Slc3a2, (C) Tob2, were made using the Integrative Genomics Viewer tool [155, 156]. Regions highlighted in gray correspond to the gene body while those in green correspond to the genomic regions where the TRs and GRs can be found. (TIF) [file pone.0220378.s002.tif]

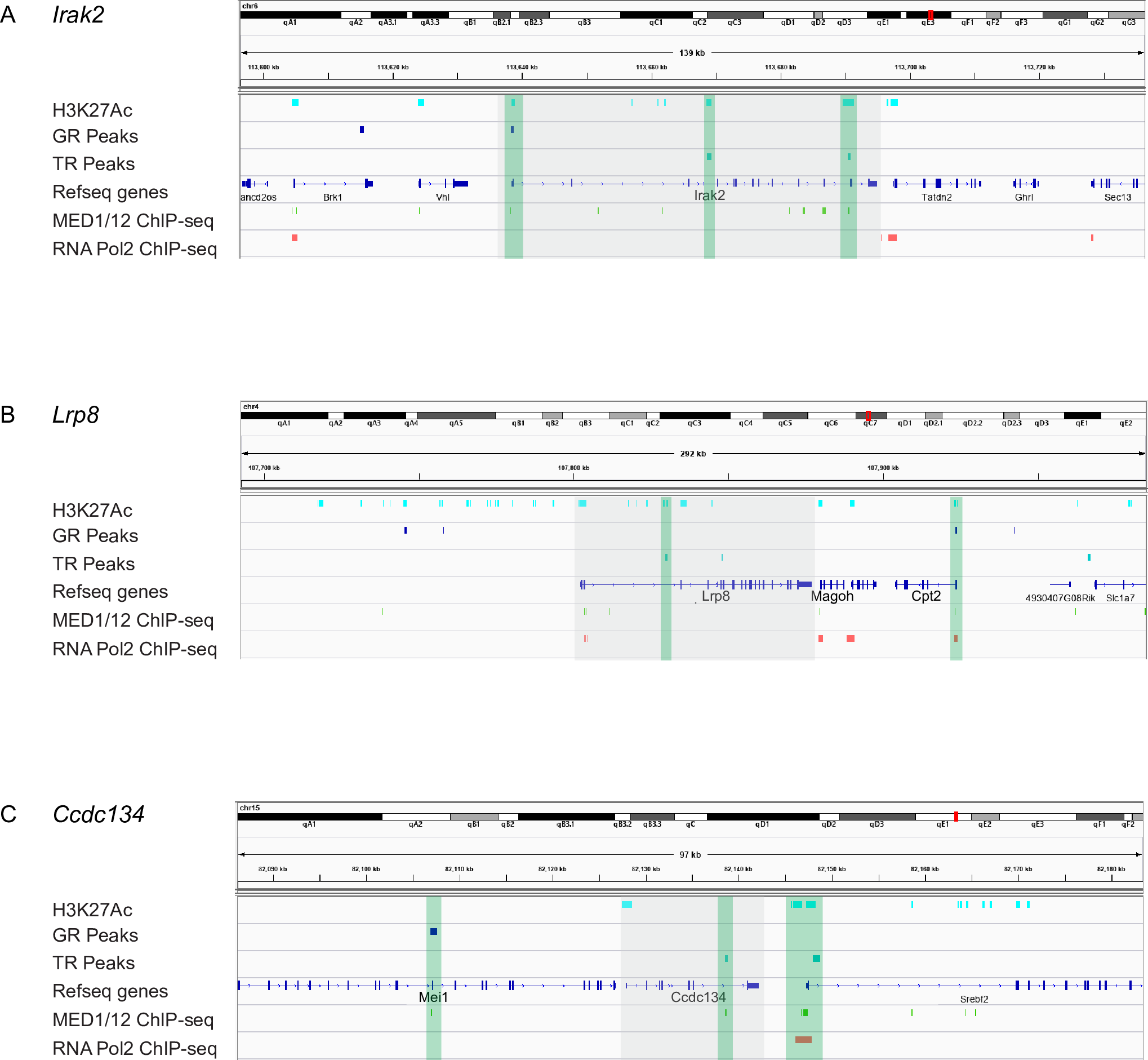

Supplement: S3 Fig — Genomic plots showing representative genes (A) Irak2, (B) Lrp8, (C) Ccdc134 were made using the Integrative Genomics Viewer tool [155, 156]. Regions highlighted in gray correspond to the gene body while those in green correspond to the genomic regions where the TRs and GRs can be found. (TIF) [file pone.0220378.s003.tif]

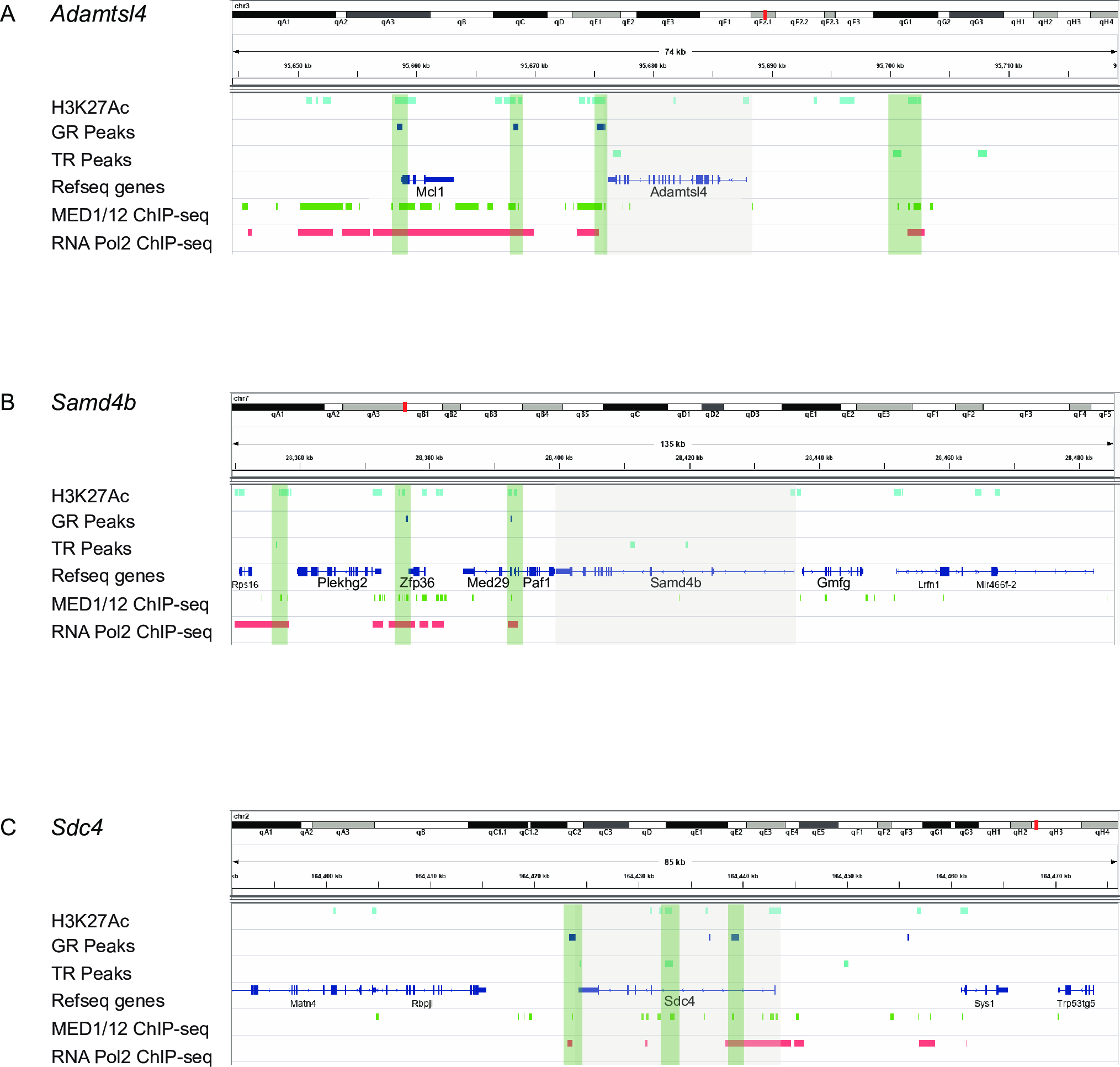

Supplement: S4 Fig — Genomic plots showing representative genes (A) Adamtsl4, (B) Samd4b, (C) Sdc4 were made using the Integrative Genomics Viewer tool [155, 156]. Regions highlighted in gray correspond to the gene body while those in green correspond to the genomic regions where the TRs and GRs can be found. (TIF) [file pone.0220378.s004.tif]

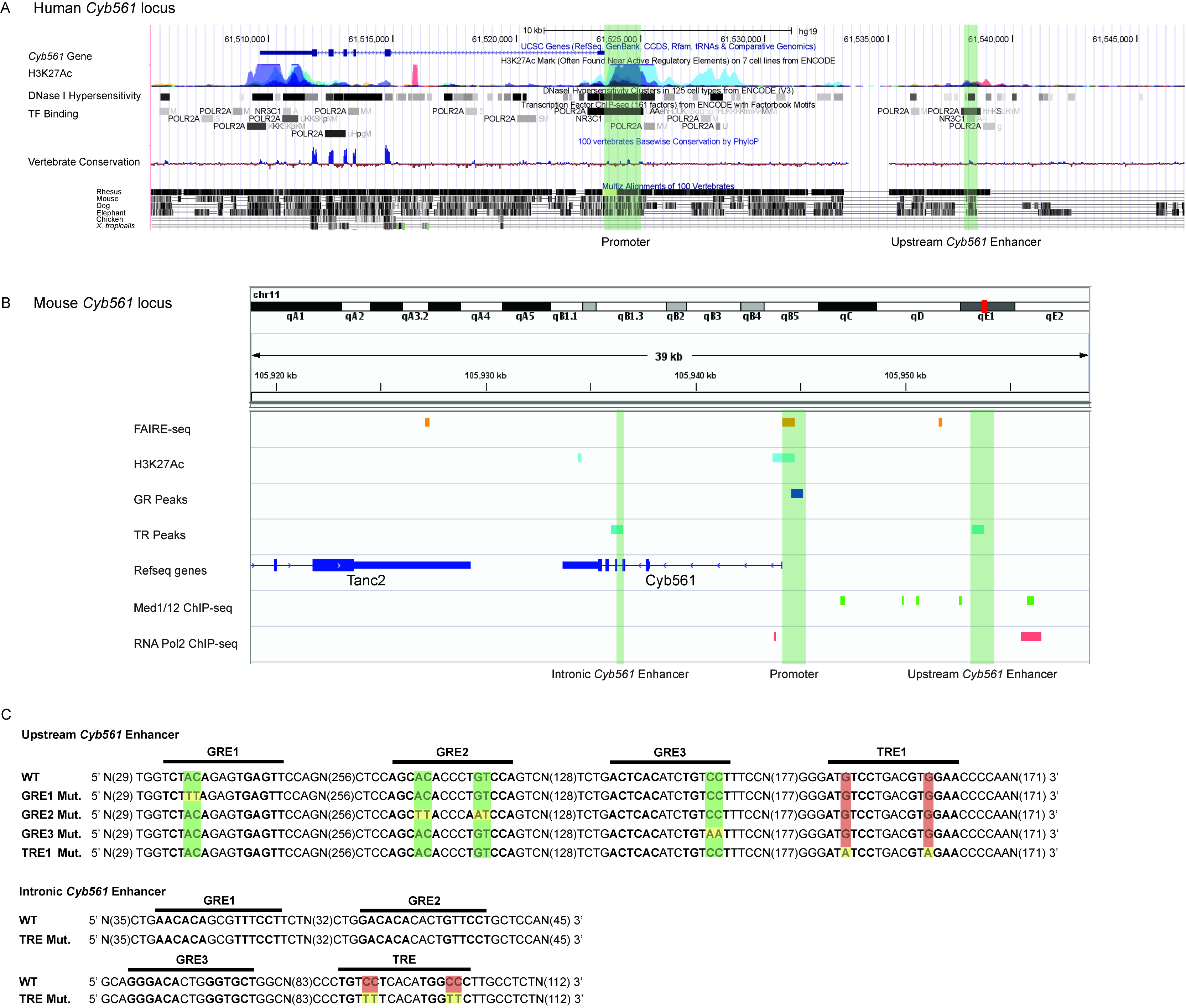

Supplement: S5 Fig — (A) Genomic plot of the human Cyb561 locus showing characteristic open chromatin marks (H3K27 hyper acetylation, DNAse I sensitivity, transcription factor binding) and vertebrate conservation plotted using the UCSC Genome Browser [157] based on the hg19 build of the human genome. (B) Genomic plot of the mouse Cyb561 locus and open chromatin marked by FAIRE-seq and H3K27Ac peaks, the Mediator complex subunits MED1 and MED12, GR, and TR peaks determined by ChIP-seq [27, 57–59]. Data were plotted using the Integrative Genomics Viewer [155, 156] on the mm10 build of the mouse genome. (C) Alignments of the WT UCE and ICE constructs with generated mutants. Conserved bases in the GREs are highlighted in green while conserved bases in the TREs are highlighted in red. Yellow highlights indicate bases which were edited in the respective mutants. (TIF) [file pone.0220378.s005.tif]

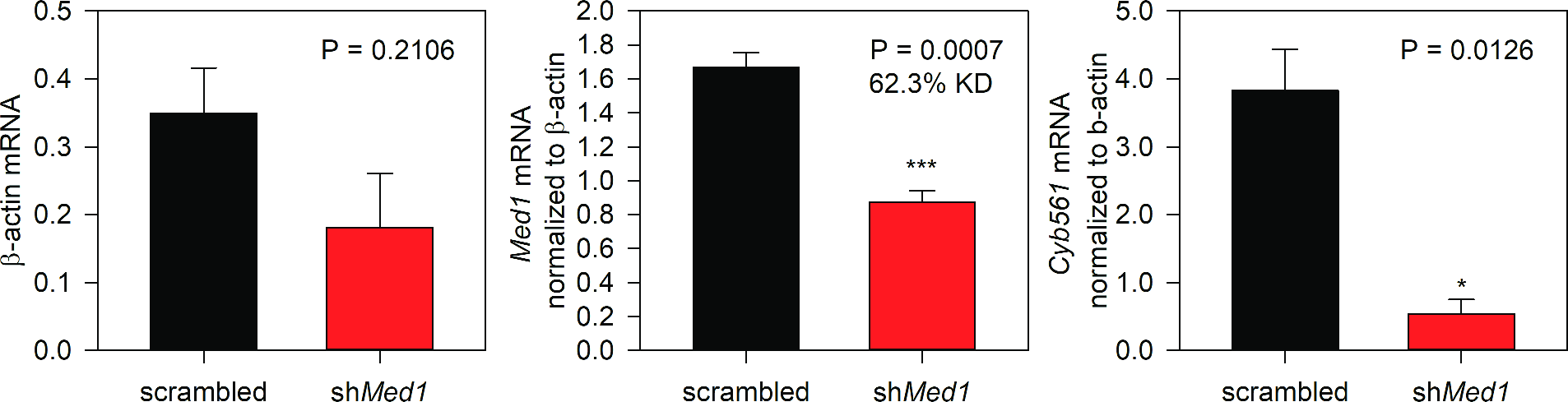

Supplement: S6 Fig — HT22 cells were stably transduced with a Med1 shRNA lentiviral construct or with a scrambled shRNA lentiviral control. Stably transduced cells were selected with 2 μg/μL puromycin. Cells were grown in selection media for three passages before harvest and RNA extraction. We measured gene expression by RTqPCR for (A) β-actin, (B) Med1, and (C) Cyb561. There was no significant effect of Med1 knockdown on the expression of the reference gene β-actin used for normalization of RNA transcripts. HT-22 cells transduced with shMed1 exhibit significantly reduced Med1 mRNA expression at 62.3% knockdown compared to the scrambled control. Basal Cyb561 expression in HT-22 cells transduced with shMed1 is significantly reduced compared to scrambled control. (TIF) [file pone.0220378.s006.tif]

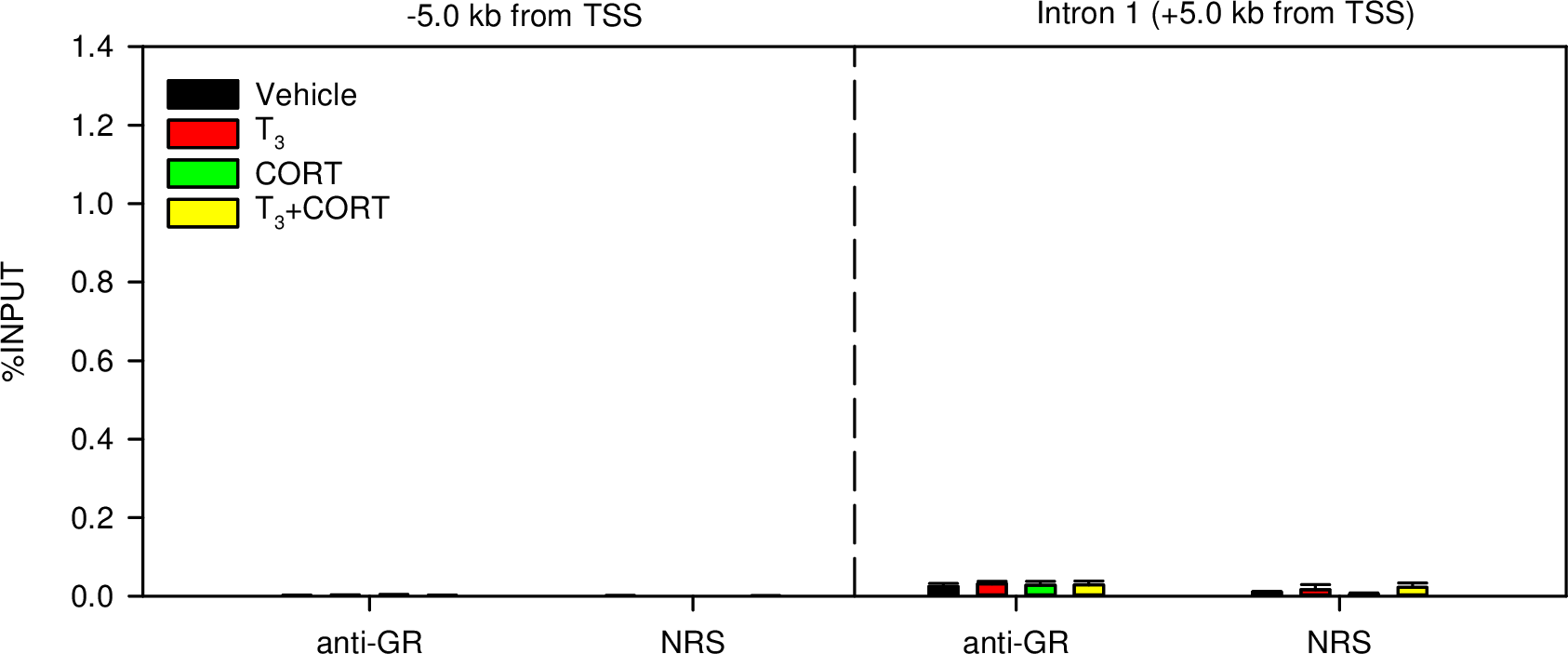

Supplement: S7 Fig — Neither the region -5.0 kb upstream of TSS nor +5.5 kb downstream of TSS (intron 1) of the Cyb561 gene exhibit GR-association when tested through ChIP qPCR. Bars represent the mean ± SEM in % input for ChIP analysis, and letters above the means indicate significant differences among treatments (means with the same letter are not significantly different; Tukey’s multiple comparison test; P < 0.05). (TIF) [file pone.0220378.s007.tif]
