# Supplementary material for: Coordinated transcriptional regulation by thyroid hormone and glucocorticoid interaction in adult mouse hippocampus-derived neuronal cells
Source: PLoS One. 2019 Jul 26;14(7):e0220378. doi: 10.1371/journal.pone.0220378 (PMC6660079; doi:10.1371/journal.pone.0220378)
Supplement: S1 Table — (DOCX) [file pone.0220378.s008.docx]

**S1 Table. Primers used for RT-qPCR.**

| **Target** | **Forward Primer** | **Reverse Primer** |
| --- | --- | --- |
| *Tas1r1* | TGATAACACTGACCACGCTG | CCTGGTACTTATCGCTGGG |
| *Dbp* | CCATGAGACTTTTGACCCTCG | TCATTGTTCTTGTACCTCCGG |
| *Ppm1h* | AACCATTCCTGTCTTCAGCTC | TGAGTGATTGCTTCCGCTAC |
| *Pdk4* | AGAGCCTGATGGATTTGGTG | CTTGAGCCATTGTAGGGACC |
| *Phlda1* | CCGTACCAACTCCAGCAC | GCACTCTTCCCACTTTCCG |
| *Egr1* | AGCGCCTTCAATCCTCAAG | TTTGGCTGGGATAACTCGTC |
| *Klf13* | CTACAGCCGCTCCGATG | CTGTTTTGCTGTGGACTTCTC |
| *Klf9* | GCACAAGTGCCCCTACAGT | TGTATGCACTCTGTAATGGGCTTT |
| *Cyb561* | TGGTGTGGGATCTTAGTCTTTG | CAAAGAAAATGTGCTGAGGGC |
| *Cyb561* eRNA | GGCCAGGAACATGTGAGTAAG | GAGTGGGAGGGTCTAATGAAG |
| *Errfi1* | TGGCCTACAATCTGAACTCCC | GACCACACTCTGCAAAGAAGT |
| *Per1* | TGTGTCAAGCAGGTTCAGG | TGTCCTGGTTTCGAAGTGTG |
| *Ppia* | GGTTCCTCCTTTCACAGAAT | AATTTCTCTCCGTAGATGGAC |
| *Β-actin* | CTAAGGCCAACCGTGAAAAG | ACCAGAGGCATACAGGGACA |
| *Med1* | ACACCAAGTGGCCTATAACAC | CACTGAAGCGAGATTCTGAGAG |
