# Supplementary material for: Coordinated transcriptional regulation by thyroid hormone and glucocorticoid interaction in adult mouse hippocampus-derived neuronal cells
Source: PLoS One. 2019 Jul 26;14(7):e0220378. doi: 10.1371/journal.pone.0220378 (PMC6660079; doi:10.1371/journal.pone.0220378)
Supplement: S2 Table — (DOCX) [file pone.0220378.s009.docx]

**S2 Table. Primers used for cloning and site-directed mutagenesis.**

| **Target** | **Forward Primer** | **Reverse Primer** |
| --- | --- | --- |
| Upstream *Cyb561* Enhancer (UCE) | GAAAGAGCTCAGGCGGATTTCTGAGTTTGAG | GTATAGATCTTCTGATGCCTGGAGGTAGAA |
| Intronic *Cyb561* Enhancer (ICE) | AAGAGCTCTCTTCGCCTTCATCATCG | AACTCGAGCCTGCACAAAGTACAGAAC |
| UCE GRE1 Mutagenesis | TTTGAGGCCAGCCTGGTCTTTAGAGTGAGTTCCAGG | CCTGGAACTCACTCTAAAGACCAGGCTGGCCTCAAA |
| UCE GRE2 Mutagenesis | CAATCCAGTCCATGCACCCCCAGGC | GGTAAGCTGGAGACAGGGTTGGGCCC |
| UCE GRE3 Mutagenesis | AGTAAGTTCTGACTCACATCTAACCTTTCCTGGTTGCCTC | GAGGCAACCAGGAAAGGTTAGATGTGAGTCAGAACTTACT |
| UCE TRE1 Mutagenesis | ACGTAGAACCCCAAGAGGAGTTGAAAGCC | CAGGATATCCCCACGGGTCTGTGCAG |
| ICE TRE Mutagenesis | CATGGTTCTTGCCTCTGCTCCAGGGT | TGAAAACAGGGCTAGTCATGGAGAAGG |
