# Supplementary material for: Coordinated transcriptional regulation by thyroid hormone and glucocorticoid interaction in adult mouse hippocampus-derived neuronal cells
Source: PLoS One. 2019 Jul 26;14(7):e0220378. doi: 10.1371/journal.pone.0220378 (PMC6660079; doi:10.1371/journal.pone.0220378)
Supplement: S3 Table — (DOCX) [file pone.0220378.s010.docx]

**S3 Table. Primers used for ChIP.**

| **Target** | **Forward Primer** | **Reverse Primer** | **Probe** |
| --- | --- | --- | --- |
| UCE | GCCAAGGAAGGAGTAAATGAA C | GATAGCGGCAATGCATCTTG | 6FAM-AGGCAACCAGGAAAGGACAGA-BHQ1 TGT |
| ICE | GGAGGAAACACTAGCCAGAAC | CTCTTCTTCTCACAGGCATCAC | 6FAM-ATCATCTGGCTGGACCAAAGCCC-BHQ1 |
| *Cyb561* Intron 1 (+5.5 kb of TSS) | CTTTCTCTGTTGGATACCCGAG | CCCCAAAACTTCCTTACCCTC | - |
| -5.0 kb of *Cyb561* TSS | GGAATAGAAACCCTAAGACACCC | AACCATAAACCTCAGGGCAG | - |
