# Supplementary material for: Coordinated transcriptional regulation by thyroid hormone and glucocorticoid interaction in adult mouse hippocampus-derived neuronal cells
Source: PLoS One. 2019 Jul 26;14(7):e0220378. doi: 10.1371/journal.pone.0220378 (PMC6660079; doi:10.1371/journal.pone.0220378)
Supplement: S4 Table — (DOCX) [file pone.0220378.s011.docx]

**S4 Table. Genes differentially regulated by T_3_.**

| **SYMBOL** | **T_3_ Fold Change** | **CORT Fold Change** | **T_3_ + CORT Fold Change** |
| --- | --- | --- | --- |
| *Klf9* | 3.09 | 2.17 | 5.52 |
| *Tas1r1* | 2.68 | 1.29 | 3.20 |
| *Cyb561* | 2.61 | 3.78 | 10.52 |
| *2310051E17Rik* | 2.23 | 2.19 | 5.25 |
| *Spon2* | 1.93 | 0.81 | 0.66 |
| *Dbp* | 1.79 | 1.50 | 2.52 |
| *C030002B11Rik/Ppm1h* | 1.74 | 1.27 | 1.48 |
| *Npr3* | 1.59 | 0.94 | 1.29 |
| *Cdon* | 1.51 | 0.79 | 1.24 |
