# Supplementary material for: Coordinated transcriptional regulation by thyroid hormone and glucocorticoid interaction in adult mouse hippocampus-derived neuronal cells
Source: PLoS One. 2019 Jul 26;14(7):e0220378. doi: 10.1371/journal.pone.0220378 (PMC6660079; doi:10.1371/journal.pone.0220378)
Supplement: S5 Table — (DOCX) [file pone.0220378.s012.docx]

**S5 Table. Top 10 genes induced and repressed by CORT.**

| **SYMBOL** | **T_3_ Fold Change** | **CORT Fold Change** | **T_3_ + CORT Fold Change** |
| --- | --- | --- | --- |
| **UP** | | | |
| *Tsc22d3* | 1.10 | 8.82 | 7.25 |
| *Pdk4* | 0.76 | 5.02 | 4.71 |
| *Per1* | 1.11 | 4.64 | 4.29 |
| *S3-12* | 1.02 | 4.48 | 2.80 |
| *Per2* | 1.15 | 4.21 | 3.92 |
| *Map3k6* | 1.05 | 3.82 | 3.10 |
| *Fam107a* | 0.99 | 3.80 | 2.78 |
| *Cyb561* | 2.61 | 3.78 | 10.52 |
| *Lcn2* | 1.01 | 3.74 | 2.82 |
| *Rn18s* | 0.95 | 3.69 | 3.65 |
| **DOWN** | | | |
| *Cyr61* | 1.06 | 0.32 | 0.52 |
| *Phlda1* | 0.97 | 0.32 | 0.35 |
| *Egr1* | 1.01 | 0.34 | 0.47 |
| *1110004P21Rik* | 0.89 | 0.41 | 0.50 |
| *A630084D02Rik* | 0.90 | 0.41 | 0.44 |
| *Ngfb* | 0.82 | 0.42 | 0.41 |
| *Sertad4* | 1.04 | 0.43 | 0.37 |
| *Rnu6* | 0.96 | 0.43 | 0.50 |
| *Paip1* | 0.92 | 0.46 | 0.48 |
| *Aldh3a1* | 0.68 | 0.50 | 0.45 |
