# Supplementary material for: Coordinated transcriptional regulation by thyroid hormone and glucocorticoid interaction in adult mouse hippocampus-derived neuronal cells
Source: PLoS One. 2019 Jul 26;14(7):e0220378. doi: 10.1371/journal.pone.0220378 (PMC6660079; doi:10.1371/journal.pone.0220378)
Supplement: S6 Table — (DOCX) [file pone.0220378.s013.docx]

**S6 Table. Top 10 genes induced and repressed by T_3_ + CORT**

| **SYMBOL** | **T_3_ Fold Change** | **CORT Fold Change** | **T_3_ + CORT Fold Change** |
| --- | --- | --- | --- |
| **UP** | | | |
| *Cyb561* | 2.61 | 3.78 | 10.52 |
| *Tsc22d3* | 1.10 | 8.82 | 7.25 |
| *Klf9* | 3.09 | 2.17 | 5.52 |
| *Pdk4* | 0.76 | 5.02 | 4.71 |
| *Per1* | 1.11 | 4.64 | 4.29 |
| *Per2* | 1.15 | 4.21 | 3.92 |
| *Ear3* | 1.06 | 2.50 | 3.79 |
| *Rn18s* | 0.95 | 3.69 | 3.65 |
| *Tas1r1* | 2.68 | 1.29 | 3.20 |
| *Map3k6* | 1.05 | 3.82 | 3.10 |
| **DOWN** | | | |
| *Phlda1* | 0.97 | 0.32 | 0.35 |
| *Ccl7* | 1.06 | 0.62 | 0.37 |
| *Sertad4* | 1.04 | 0.43 | 0.37 |
| *Ngfb* | 0.89 | 0.43 | 0.38 |
| *A630084D02Rik* | 0.90 | 0.41 | 0.44 |
| *Aldh3a1* | 0.68 | 0.50 | 0.45 |
| *Egr1* | 1.01 | 0.34 | 0.47 |
| *Fgf7* | 1.04 | 0.52 | 0.47 |
| *Vcam1* | 0.88 | 0.57 | 0.47 |
| *Angptl4* | 1.15 | 0.72 | 0.48 |
