# Supplementary material for: Coordinated transcriptional regulation by thyroid hormone and glucocorticoid interaction in adult mouse hippocampus-derived neuronal cells
Source: PLoS One. 2019 Jul 26;14(7):e0220378. doi: 10.1371/journal.pone.0220378 (PMC6660079; doi:10.1371/journal.pone.0220378)
Supplement: S8 Table — (DOCX) [file pone.0220378.s015.docx]

**S8 Table. *In silico* analysis of synergistically regulated genes for GR and TR peak binding within 1kb of MED1/12 peaks.**

|  | **T_3_ Fold Change** | **CORT Fold Change** | **T_3_ + CORT Fold Change** |
| --- | --- | --- | --- |
| TR Only | | | |
| Angptl4 | 1.15 | 0.72 | 0.48 |
| Arfip2 | 1.00 | 1.43 | 1.57 |
| C77080 | 0.94 | 0.79 | 0.62 |
| Calr | 0.95 | 1.40 | 1.54 |
| Clip2 | 1.07 | 0.70 | 0.64 |
| Cyb561 | 2.61 | 3.78 | 10.52 |
| Cyb5r3 | 1.03 | 1.49 | 1.57 |
| Hspg2 | 1.28 | 0.76 | 0.66 |
| Plekhf1 | 1.08 | 1.49 | 1.56 |
| Rab34 | 1.02 | 1.42 | 1.62 |
| Rnf135 | 1.04 | 1.26 | 1.51 |
| Sh3gl1 | 1.17 | 1.45 | 1.58 |
| Smarcd2 | 1.09 | 1.47 | 1.53 |
| Tead2 | 0.94 | 0.68 | 0.62 |
| Tomm6 | 0.96 | 0.67 | 0.62 |
| Trim25 | 1.06 | 1.44 | 1.50 |
| Uba1 | 1.02 | 1.36 | 1.52 |
| Uck1 | 1.06 | 1.42 | 1.57 |
| Vrk3 | 1.08 | 1.48 | 1.59 |
| GR Only | | | |
| BC028528 | 0.84 | 0.74 | 0.66 |
| Cope | 1.01 | 1.44 | 1.58 |
| Fam152b | 1.10 | 1.30 | 1.51 |
| Dusp8 | 1.10 | 0.78 | 0.63 |
| Eps8 | 1.08 | 0.72 | 0.63 |
| Errfi1 | 1.21 | 1.46 | 2.10 |
| Id2 | 0.90 | 1.27 | 1.59 |
| Lims2 | 1.07 | 1.89 | 2.37 |
| Med16 | 1.03 | 0.74 | 0.65 |
| Plekha1 | 1.48 | 1.24 | 1.54 |
| Psme2 | 0.91 | 0.71 | 0.67 |
| Rnf135 | 1.04 | 1.26 | 1.51 |
| D15Ertd682e | 0.95 | 1.39 | 1.55 |
| Spnb2 | 0.88 | 0.80 | 0.66 |
| Trim56 | 0.95 | 0.75 | 0.66 |
| Ttc3 | 0.98 | 0.77 | 0.63 |
| Vat1 | 1.01 | 0.83 | 0.66 |
| Vps26b | 1.03 | 1.45 | 1.54 |
| Zfhx3 | 1.07 | 0.79 | 0.55 |
| Zfp292 | 0.88 | 0.68 | 0.66 |
| TR and GR | | | |
| Actb | 0.93 | 0.63 | 0.58 |
| Accn2 | 1.10 | 0.74 | 0.62 |
| Jmjd3 | 1.23 | 1.30 | 1.54 |
| Klf9 | 3.09 | 2.17 | 5.52 |
| Nfic | 1.02 | 0.76 | 0.63 |
| Ppp1r10 | 1.02 | 1.49 | 1.53 |
| Psmd2 | 1.01 | 1.34 | 1.58 |
| Slc1a5 | 1.05 | 1.44 | 1.56 |
| Slc3a2 | 1.20 | 1.48 | 1.73 |
| Tob2 | 1.03 | 1.42 | 1.53 |
| Tspan4 | 1.18 | 1.32 | 1.57 |
| Uhrf1 | 0.90 | 1.42 | 1.53 |
| Cfl1 | 1.16 | 0.71 | 0.63 |
