# Supplementary material for: Coordinated transcriptional regulation by thyroid hormone and glucocorticoid interaction in adult mouse hippocampus-derived neuronal cells
Source: PLoS One. 2019 Jul 26;14(7):e0220378. doi: 10.1371/journal.pone.0220378 (PMC6660079; doi:10.1371/journal.pone.0220378)
Supplement: S11 Table — (DOCX) [file pone.0220378.s018.docx]

**S11 Table. *In silico* analysis of genes whose CORT-response is altered by T_3_ for GR and TR peak binding within 1kb of MED1/12 peaks.**

|  | **T_3_ Fold Change** | **CORT Fold Change** | **T_3_ + CORT Fold Change** |
| --- | --- | --- | --- |
| TR Peaks Only | | | |
| Sphk1 | 1.05 | 3.24 | 2.27 |
| Ier3 | 0.85 | 0.58 | 0.66 |
| Irak2 | 1.05 | 0.53 | 0.66 |
| GR Peaks Only | | | |
| Lims2 | 1.08 | 2.36 | 2.91 |
| Sesn1 | 0.95 | 3.35 | 2.77 |
| Cyr61 | 1.06 | 0.32 | 0.52 |
| TR and GR | | | |
| Ccdc134 | 0.82 | 2.92 | 1.72 |
