# Supplementary material for: Coordinated transcriptional regulation by thyroid hormone and glucocorticoid interaction in adult mouse hippocampus-derived neuronal cells
Source: PLoS One. 2019 Jul 26;14(7):e0220378. doi: 10.1371/journal.pone.0220378 (PMC6660079; doi:10.1371/journal.pone.0220378)
Supplement: S12 Table — (DOCX) [file pone.0220378.s019.docx]

**S12 Table. *In silico* analysis of genes whose CORT-response is altered by T_3_ for GR and TR peak binding within 1kb of RNA Pol2.**

|  | **T_3_ Fold Change** | **CORT Fold Change** | **T_3_ + CORT Fold Change** |
| --- | --- | --- | --- |
| TR Peaks Only | | | |
| Ccdc134 | 0.82 | 2.92 | 1.72 |
| GR Peaks Only | | | |
| Rasl11b | 0.85 | 2.54 | 2.02 |
| Ier3 | 0.85 | 0.58 | 0.66 |
