# Supplementary material for: Coordinated transcriptional regulation by thyroid hormone and glucocorticoid interaction in adult mouse hippocampus-derived neuronal cells
Source: PLoS One. 2019 Jul 26;14(7):e0220378. doi: 10.1371/journal.pone.0220378 (PMC6660079; doi:10.1371/journal.pone.0220378)
Supplement: S15 Table — (DOCX) [file pone.0220378.s022.docx]

**S15 Table. *In silico* analysis of CORT-responsive genes that lose CORT response with T_3_ for GR and TR peak binding within 1kb of RNA Pol2 peaks.**

|  | **T_3_ Fold Change** | **CORT Fold Change** | **T_3_+CORT Fold Change** |
| --- | --- | --- | --- |
| TR Peaks Only | | | |
| Add1 | 1.06 | 1.61 | 1.45 |
| Hist2h2ab | 0.86 | 1.60 | 1.27 |
| Il4i1 | 1.19 | 1.54 | 1.35 |
| Pgpep1 | 1.13 | 1.60 | 1.09 |
| Slc35a3 | 0.98 | 0.65 | 0.70 |
| Sult1a1 | 0.93 | 1.55 | 1.33 |
| Tardbp | 0.83 | 0.66 | 0.75 |
| Tmem50a | 0.93 | 1.51 | 1.35 |
| GR Peaks Only | | | |
| Asna1 | 1.07 | 0.64 | 0.82 |
| Bhlhb2 | 1.15 | 1.76 | 1.42 |
| Fndc7 | 0.96 | 1.58 | 1.16 |
| Klf2 | 1.07 | 1.52 | 1.31 |
| Leng9 | 1.00 | 1.55 | 1.37 |
| Rin2 | 1.10 | 1.61 | 1.40 |
| TR and GR | | | |
| Actb | 1.25 | 1.64 | 1.44 |
| Adamtsl4 | 1.08 | 1.68 | 1.39 |
| Ier5l | 1.03 | 1.62 | 1.43 |
| Plekhf1 | 1.06 | 1.65 | 1.49 |
| Samd4b | 1.04 | 0.67 | 0.72 |
| Sdc4 | 1.14 | 1.59 | 1.36 |
