# Supplementary material for: Coordinated transcriptional regulation by thyroid hormone and glucocorticoid interaction in adult mouse hippocampus-derived neuronal cells
Source: PLoS One. 2019 Jul 26;14(7):e0220378. doi: 10.1371/journal.pone.0220378 (PMC6660079; doi:10.1371/journal.pone.0220378)
Supplement: S16 Table — (DOCX) [file pone.0220378.s023.docx]

**S16 Table. Summary of differential expression analysis results for microarray and RT-qPCR validation.**

|  |  | **Microarray** | | | **RT-qPCR** | | |
| --- | --- | --- | --- | --- | --- | --- | --- |
| **SYMBOL** | **DIRECTION** | **T_3_-FC** | **CORT-FC** | **T_3_ + CORT-FC** | **T_3_-FC** | **CORT-FC** | **T_3_ + CORT-FC** |
| **T_3_** | | | | | | | |
| Tas1r1 | UP | 2.68 | 1.29 | 3.20 | 4.96 | 1.49 | 6.80 |
| Dbp | UP | 1.79 | 1.50 | 2.52 | 1.53 | 0.55 | 1.66 |
| C030002B11Rik/Ppm1h | UP | 1.74 | 1.27 | 1.48 | 2.86 | 0.69 | 1.15 |
| **CORT** | | | | | | | |
| Pdk4 | UP | 0.76 | 5.02 | 4.71 | 1.25 | 7.01 | 3.46 |
| Phlda1 | DOWN | 0.97 | 0.32 | 0.35 | 1.41 | 0.65 | 0.73 |
| Egr1 | DOWN | 1.01 | 0.34 | 0.47 | 0.90 | 0.35 | 0.47 |
| Klf13 | UP | 1.03 | 1.72 | 1.61 | 0.71 | 2.45 | 2.37 |
| Cyr61 | DOWN | 1.06 | 0.32 | 0.52 | 0.98 | 0.43 | 0.24 |
| **T_3_ + CORT** | | | | | | | |
| Klf9 | UP | 3.09 | 2.17 | 5.52 | 1.80 | 1.48 | 5.65 |
| Cyb561 | UP | 2.61 | 3.78 | 10.52 | 7.67 | 4.37 | 14.94 |
| Per1 | UP | 1.11 | 4.64 | 4.29 | 1.06 | 11.70 | 11.43 |
| Errfi1 | UP | 1.21 | 1.46 | 2.10 | 1.07 | 2.28 | 2.48 |
